# *Pseudomonas aeruginosa* Uses Dihydrolipoamide Dehydrogenase (Lpd) to Bind to the Human Terminal Pathway Regulators Vitronectin and Clusterin to Inhibit Terminal Pathway Complement Attack

**DOI:** 10.1371/journal.pone.0137630

**Published:** 2015-09-14

**Authors:** Teresia Hallström, Melanie Uhde, Birendra Singh, Christine Skerka, Kristian Riesbeck, Peter F. Zipfel

**Affiliations:** 1 Department of Infection Biology, Leibniz Institute for Natural Product Research and Infection Biology, Hans Knoell Institute, Jena, Germany; 2 Medical Microbiology, Department of Translational Medicine Malmö, Lund University, Skåne University Hospital, Malmö, Sweden; 3 Faculty of Biology, Friedrich Schiller University, Jena, Germany; University of Kentucky College of Medicine, UNITED STATES

## Abstract

The opportunistic human pathogen *Pseudomonas aeruginosa* controls host innate immune and complement attack. Here we identify Dihydrolipoamide dehydrogenase (Lpd), a 57 kDa moonlighting protein, as the first *P*. *aeruginosa* protein that binds the two human terminal pathway inhibitors vitronectin and clusterin. Both human regulators when bound to the bacterium inhibited effector function of the terminal complement, blocked C5b-9 deposition and protected the bacterium from complement damage. *P*. *aeruginosa* when challenged with complement active human serum depleted from vitronectin was severely damaged and bacterial survival was reduced by over 50%. Similarly, when in human serum clusterin was blocked by a mAb, bacterial survival was reduced by 44%. Thus, demonstrating that *Pseudomonas* benefits from attachment of each human regulator and controls complement attack. The Lpd binding site in vitronectin was localized to the C-terminal region, i.e. to residues 354–363. Thus, Lpd of *P*. *aeruginosa* is a surface exposed moonlighting protein that binds two human terminal pathway inhibitors, vitronectin and clusterin and each human inhibitor when attached protected the bacterial pathogen from the action of the terminal complement pathway. Our results showed insights into the important function of Lpd as a complement regulator binding protein that might play an important role in virulence of *P*. *aeruginosa*.

## Introduction

*Pseudomonas aeruginosa* is an opportunistic, Gram-negative pathogenic bacterium that can reside in the human organism as a harmless commensal but can also cause a broad range of acute and chronic diseases [1]. *Pseudomonas aeruginosa* is a major cause of chronic lung infections in cystic fibrosis patients and this bacterium is often responsible for hospital-acquired infections, such as pneumonia and septicemia [2,3]. Especially immuno-compromised individuals, such as HIV patients and cancer patients are at risk for infections with this pathogen [4]. *P*. *aeruginosa* is one of the leading causes of ventilator-associated pneumonia [5]. Antibiotic resistance, biofilm formation and the production of multiple virulence factors make *P*. *aeruginosa* infections difficult to treat and suitable drugs controlling infections caused by this pathogenic bacterium need to be developed.

*P*. *aeruginosa* expresses both surface exposed and secreted virulence factors e.g. Dihydrolipoamide dehydrogenase (Lpd), exotoxins as well as proteases [6–10]. Lpd is a 57 kDa surface exposed moonlighting protein of *Pseudomonas*, which is also present in the cytoplasm [9,11,12]. In the cytoplasm, Lpd is a component of a multi-enzyme pyruvate dehydrogenase complex, which catalyzes the electron transfer between pyridine nucleotides and disulfide components. The bacterial surface protein Lpd binds several human plasma proteins and complement regulators, including Factor H, Factor H-like protein 1 (FHL-1), complement Factor H related protein 1 (CFHR1) and the proteolytic proenzyme plasminogen [9]. Attached to Lpd, these human plasma proteins block complement activation, complement cascade progression and thus protect the bacterium from complement attack and the production of complement effector proteins [9].

Upon infection of a human organism, infectious microbes are immediately confronted and attacked by the host complement system, which forms the first defense line of host innate immunity [13]. The activated complement cascade generates effector components, like the antimicrobial and anaphylactic peptides C3a and C5a, deposits C3b onto target surfaces (opsonisation) and forms the terminal complement complex (TCC) [14]. Complement is initiated by three major pathways, the alternative, the classical and the lectin pathway and each pathway initiates a series of tightly regulated reactions that all merge on the level of C3 and generate C3 convertases and an amplification loop [15,16]. Cascade progression generates a C5 convertase that cleaves C5, into C5a and C5b [16]. Surface attached C5b initiates the terminal pathway. Thereafter, C6 and C7 bind to C5b, form a short lived C5b-7 complex, which can attach and insert into a target membranes [17]. C8 and C9 bind to surface attached C5b-7, allow assembly and formation of the C5b-9 complex (C5b-9/TCC) forms a lytic pore and allows polymerization of C9 [18]. C5b-9 when inserted into the target membrane changes the osmotic pressure and causes target lysis. When activation and cascade progression are not properly controlled, the complement cascade is further amplified and in consequence toxic effector components, including anaphylatoxins, inflammatory mediators, antimicrobial products and TCC are generated [14]. In general terms human complement attack is targeted to foreign, infectious microbes and to modified self-components [13,14] and intact host cells and tissues are protected from the damaging effects of the activated complement system [19]. Host cells use a series of complement inhibitors and regulators that are either integral membrane proteins or soluble proteins that a distributed in plasma and body fluids and that are attached to a self-surface in order to efficiently control and block complement activation and action [20].

In order to survive and to establish an infection, any pathogenic microbe must evade host complement and innate immune attack [13]. Acquisition of human plasma proteins and complement regulators is a general, common and important evasion strategy used by *P*. *aeruginosa* and also by many other pathogenic microbes [13]. *P*. *aeruginosa* binds the complement regulators Factor H, FHL-1 and CFHR1 and the regulators attached to the bacterial surface inhibit complement activation and cascade progression [9,21]. Thereby they block the generation of complement effector proteins, like surface opsonization with C3b and formation of TCC and thus increase survival of the pathogenic bacterium in human serum [9,21]. Lpd and Elongation factor Tu (Tuf) are two *P*. *aeruginosa* Factor H, FHL-1 and CFHR1 binding proteins. In addition, the secreted *P*. *aeruginosa* proteases elastase and alkaline protease degrade and inactivate the human complement components C1q, C2 and C3 [6,10]. Degradation of C3 and C3b reduce opsonisation and bacterial phagocytosis by human neutrophils and thereby may increase the bacterial survival in the host [10].

Vitronectin is a central inhibitor of the terminal complement pathway and an important human adhesion protein [22]. Vitronectin circulates in plasma at concentrations, ranging from 200 to 700 μg/ml and in two variants of 75 and one of 65 kDa [22]. Vitronectin binds to the meta-stable membrane attachment site of the C5b-7 complex, thereby blocks insertion into the target membrane and inhibits C9 polymerization and attachment of C9 [23–25]. Clusterin as the second soluble human terminal pathway regulator circulates in human plasma at a concentration of ∼150–540 μg/ml [26]. Clusterin is a disulfide-linked heterodimer containing one α-chain and one β-chain of each 40 kDa [27]. Clusterin binds to C5b-6, blocks membrane insertion of C5b-7, inhibits the formation of TCC and also C9 assembly [27,28]. Both vitronectin and clusterin are pleiotropic proteins that have a major role in cell matrix interaction [22,24,29–31].

Many pathogenic microbes bind and exploit human vitronectin, and uses surface bound regulator for immune escape and for interaction with host surfaces [22]. Known pathogens, which utilize vitronectin and the identified microbial vitronectin binding proteins, are *P*. *aeruginosa*, *Haemophilus influenzae* (Hsf and Protein E), *Haemophilus ducreyi* (DsrA), *Moraxella catarrhalis* (UspA2), *Neisseria meningitidis* (Msf) and *Neisseria gonorrhoeae* [32–36]. Vitronectin attached to the microbial surface blocks complement attack and increases survival of the pathogen in human serum. Pathogenic microbes also use vitronectin for ECM binding and for adhesion to human cells, as shown for *Streptococcus pyogenes*, *Staphylococcus epidermidis*, *Staphylococcus aureus* and *Enterococcus faecalis* [37–41]. Utilization of human vitronectin for immune escape is better understood and at present very limited information is available about pathogenic microbes that bind clusterin, the related human TCC inhibitor. So far a few pathogenic microbes have been identified that bind clusterin, including *S*. *pyogenes*, *S*. *aureus*, *S*. *epidermidis* and *Paracoccidioides brasiliensis* and also dengue virus and herpes simplex virus [42–45]. However, the function of bound clusterin is at present unclear.

Given the multifunctional role of single microbial moonlighting and immune evasion proteins, we asked whether Lpd from *P*. *aeruginosa* has additional roles in innate immune escape. Therefore we searched for additional human ligands that attach to this microbial protein. Here we identify Lpd as the first vitronectin and clusterin binding protein of *P*. *aeruginosa*. Both human complement regulators when attached to surface exposed Lpd, blocked TCC formation and thus contribute to innate immune escape of *P*. *aeruginosa*.

## Material and Methods

### Bacterial strains and culture conditions

*P*. *aeruginosa* strains SG137, American Type Culture Collection (ATCC) 27853, National Collection of Type Cultures (NCTC) 10662, PA01 and various clinical isolates were routinely cultured in enriched Nutrient Broth (NB) (Serva) at 37°C [9,21]. Twelve clinical isolates were derived from patients with different diseases. Cultures were grown to an OD _600_ ≈1.0. Transformed *Escherichia coli* M15 expressing Lpd were grown in Luria Bertani broth supplemented with 25 μg/ml kanamycin and 10 μg/ml carbenicillin.

### Expression of recombinant proteins

Recombinant Lpd was expressed in *Escherichia coli* as described [9]. The *lpd* gene (GenBank accession no. Q9I3D1) was amplified from genomic DNA of *P*. *aeruginosa* strain PAO1 by PCR using Phusion High Fidelity DNA Polymerase (Finnzymes, Espoo, Finland) or HotStarTaq DNA Polymerase (Qiagen) with specific primers (Table 1). The Lpd deletion mutants (aa 1–382, 1–287, 1–160, 141–478 and 264–478) were expressed using the *E*. *coli* pET200/D-TOPO expression system (Invitrogen). Bacteria were grown to an OD_600_ of ~0.6 and expression of deletion mutants was induced by 1mM Isopropyl-β-D-thiogalactopyranosid for 3 h at 37°C. Bacteria were centrifuged at 4700 rpm for 20 min at RT and the pellets were resuspended in Buffer A (10 mM Na_2_HPO_4_, 10 mM NaH_2_PO_4_, 10 mM Imidazol and 500 mM NaCl, pH 7.4) and sonicated. After sonication, the suspensions were centrifuged at 4700 rpm for 20 min. Thereafter, the supernatants were centrifuged at 14 000 rpm for 20 min at 4°C. After centrifugation, the supernatants were subjected to Äkta purification according to the manufacturer’s protocol. All recombinant proteins were purified by nickel affinity chromatography using HisTrap chelating columns in a Äkta fast protein liquid chromatography system (GE Healthcare, Freiburg, Germany). Recombinant fragments of human vitronectin (aa 80–396, 80–379, 80–373, 80–363, 80–353, 80–339, 80–320, 80–229) were expressed as previously described [36]. Briefly, HEK293T cells were grown in three triple flasks (Nunc) to 80% confluence using advanced DMEM supplemented with penicillin (100 U/μl) and streptomycin (100 μg/ml) and 1% FCS in 37°C with 5% CO_2_. Transfected cells were incubated for 3 days at 37°C with 5% CO_2_ followed by harvest of the supernatant. Similar volume of advanced DMEM was once again added to the cells and the procedure was repeated after 3 days. His-tagged vitronectin was secreted into the medium that was purified by Ni-NTA chromatography.

**Table 1:** Primer used for the Lpd deletion mutants.

Lpd deletion mutant	Primer	Sequence (5´to 3´)
1–382	Fw	CACCATGAGCCAGAAATTCGACGT
1–382	Rev	TCATTTCTCGAACTGAGGATGTGACCAAGCAGAGACGTTGACTTCGACGC
1–287	Fw	CACCATGAGCCAGAAATTCGACGT
1–287	Rev	TCACAGCAGGTCGGTGGTCA
1–160	Fw	CACCATGAGCCAGAAATTCGACGT
-160	Rev	TCATTTCTCGAACTGAGGATGTGACCAAGCAGACGGCGGGATCTCCACCG
141–478	Fw	CACCGTCCTGGAAGCCGAGAACGT
141–478	Rev	TCAGCGCTTCTTGCGGTTGG
264–478	Fw	CACCGCCGACAAGCTGATCGTCGCGG
264–478	Rev	TCAGCGCTTCTTGCGGTTGG

### Antibodies

Polyclonal Dihydrolipoamide dehydrogenase (Lpd) antiserum was raised by immunizing rabbits intramuscularly with 200 g of purified recombinant Lpd emulsified in Freund's complete adjuvant (FCA) (Difco and BD Biosciences, Heidelberg, Germany) and boosted on days 18 and 36 with the same dose of protein in IFA. Blood was drawn 3 weeks later. Monoclonal mouse anti-human clusterin was purchased from TecoMedical (Bünde, Germany) and monoclonal mouse anti-human vitronectin 58–1 from Abcam (Cambridge, UK). C5b-9 deposition was assayed using monoclonal mouse anti-human C5b-9 (Dako). HRP-conjugated rabbit anti-goat, HRP-conjugated goat anti-mouse and HRP-conjugated swine anti-rabbit were obtained from Dako (Glostrup, Denmark). Polyclonal rabbit anti-human vitronectin and polyclonal goat anti-Factor H were obtained from Complement Technology (Tyler, TE) and polyclonal goat anti-plasminogen from Acris Antibodies GmbH (Herford, Germany).

### Generation of vitronectin depleted serum

Vitronectin depleted human serum (HSΔ*Vn*), which retained complement activity was generated. A total of 100 μl mix of protein A-Sepharose and protein G-Sepharose (GE Healthcare, Freiburg, Germany) was incubated with polyclonal rabbit anti-vitronectin serum (200 μl) plus 300 μl Dulbecco’s PBS (DPBS) buffer (Lonza) overnight at 4°C on a shaker. Unbound vitronectin was removed by washing three times with DPBS. Thereafter, active NHS (500 μl) was added and the mixture was incubated for 45 min at 4°C. Next, the serum was collected by centrifugation. Depletion of vitronectin was confirmed by Western blotting (Supplementary data).

### Serum resistance assay

NHS was collected from five healthy individuals, pooled and stored at -80°C until use. The ethical committee of the Friedrich Schiller University in Jena, Germany has approved the study. The *P*. *aeruginosa* strain SG137 was grown to an OD_600_ ∼ 1.0 and diluted in GVB++ buffer (Complement Technology, Tyler, Texas). Bacteria (10^4^/sample) were incubated in NHS (10%) or complement active vitronectin depleted NHS (10%) in a final volume of 100 μl at 37°C. At time point 0 and after 15 min, 10 μl aliquots were removed and spread onto NB agar plates. After 18 h of incubation at 37°C, CFU were determined. To assay the effect of clusterin in survival of *P*. *aeruginosa*, strain SG137 was incubated in 1% NHS together with anti-clusterin mAb (1:50) or mouse IgG (1:50) or in 1% heat-inactivated NHS (HiNHS) (30 min at 56°C) in a final volume of 100 μl at 37°C. At time point 0 and after 30 min, 10 μl aliquots were removed and spread onto NB agar plates. After 18 h of incubation at 37°C, CFU were determined.

### Flow cytometry

The binding of monomeric vitronectin (Corning) and clusterin (BioVision) purified from human plasma to *P*. *aeruginosa* were analyzed by flow cytometry. The *P*. *aeruginosa* strain SG137 was grown to an OD_600_ ≈1.0 and washed once in PBS-BSA, then bacteria (10^8^/sample) were incubated with vitronectin (10–50 μg/ml) or clusterin (5–25 μg/ml) for 45 min at RT. Bacteria were washed and incubated for 30 min with rabbit anti-human vitronectin pAb (1:1000) or anti-human clusterin mAb (1:400) followed by Alexa fluor 488-conjugated anti-rabbit pAb (1:200) or Alexa fluor 647-conjugated anti-mouse pAb. After washings the binding was analyzed by flow cytometry (FACScan LRII, Becton-Dickinson, Mountain View, California, USA). All incubations were kept in PBS-BSA and primary and secondary antibodies were added separately as a negative control.

### ELISA

Microtiter plates (F96 Maxisorb or Polysorb, Nunc-Immuno Module) were coated with live bacteria (0.5x10^7^/well) for 1 h at 37°C, with Lpd (5 μg/ml), gelatin (5 μg/ml) or Lpd deletion mutants (13.3 nM) over night at 4°C. The plates were washed four times with PBS containing 0.1% Tween 20 (PBS-Tw) and blocked for 1 h at room temperature (RT) with PBS supplemented with 2% BSA (PBS-BSA) or blocking buffer I (Applichem). After washings, the plates were incubated for 1 h at RT with vitronectin (0.01–12.5 μg/ml), the various recombinant vitronectin constructs (5 μg/ml), clusterin (0.01–20 μg/ml), Factor H (Complement Technology, Tyler, TE) (1–40 μg/ml) or plasminogen (Chromogenix, Milano, Italy) (0.5–20 μg/ml). Thereafter, the wells were washed and incubated with a rabbit anti-human vitronectin (1:1000), mouse anti-vitronectin (1:1000), mouse anti-human clusterin (1:1000), goat anti-human Factor H (1:1000), goat anti-plasminogen (1:1000) or Lpd antiserum (1:1000) followed by HRP-conjugated swine anti-rabbit (1:2500), rabbit anti-mouse (1:2500) or rabbit anti-goat (1:2500). The reaction was developed with 1,2-phenylenediamine dihydrochloride (OPD, DakoCytomation, Glostrup, Denmark) and the absorbance was measured at 492 nm. In the competition assay, the effect of heparin (10–5000 μg/ml) on vitronectin (5 μg/ml) binding to immobilized *P*. *aeruginosa* (0.5x10^7^/well) or Lpd (5 μg/ml) was analyzed.

### Serum binding assay

The binding of vitronectin or clusterin directly from NHS to *P*. *aeruginosa* was assayed in a serum-binding assay. Bacteria (10^9^) were incubated with NHS (10%) and buffer (100 mM NaCl, 50 mM Tris-HCl, pH 7.4) for 1 h at 37°C. To remove unbound proteins, bacteria were washed 5 times with the same buffer. Thereafter, the bacterial pellets were resuspended in 50 μl of 0.1% Triton X-100 (Darmstadt, Germany) and protease inhibitors (Complete, Roche, Mannheim, Germany). After 30 min incubation at 4°C, bacteria were centrifuged and the supernatants were subjected to SDS-PAGE, transferred to a membrane and analyzed by Western blotting using a rabbit anti-vitronectin (1:1000) or mouse anti-human clusterin (1:1000) for 1 h followed by HRP-conjugated swine anti-rabbit (1:2500) or HRP-conjugated goat anti-mouse (1:2500) for 40 min. Development was performed with ECL Western blotting detection reagents (Applichem).

### Ligand blotting

For ligand blotting, purified Lpd (3 μg) and BSA (3 μg) were separated by 10% SDS-PAGE and either visualized by silver staining or transferred to a nitrocellulose membrane at semi-dry conditions. After transfer, the membranes were blocked with PBS supplemented with 1% BSA and 4% milk powder for 1 h at RT and thereafter incubated with vitronectin (20 μg/ml) or clusterin (20 μg/ml) for 1 h at RT. Bound vitronectin or clusterin was detected with a rabbit anti-vitronectin (1:1000) or mouse anti-clusterin (1:1000) for 1 h followed by HRP-conjugated swine anti-rabbit (1:2500) or HRP-conjugated goat anti-mouse (1:2500) for 40 min. Development was performed with ECL Western blotting detection reagents (Applichem).

### Microscale thermophoresis (MST)

The binding of vitronectin and clusterin to Lpd were evaluated in fluid phase using MST. Human vitronectin at a concentration of 13.3 μM and clusterin at a concentration of 6.65 μM were labeled with NT-647 RED-NHS (NanoTemper technologies, Munich, Germany). NT-647-labeled vitronectin (50 nM) or clusterin (25 nM) was mixed 1:1 with Lpd at 1:2 dilutions ranging from 0.001 to 50 μM and the samples were diluted in MST buffer (50 mM Tris-HCl, pH 7.6, 150 mM NaCl, 10 mM MgCl_2_ and 0.05% Tween-20). Thermophoresis was measured at 50% LED power and 80% MST power for 30 s in a Monolith NT.115 instrument (NanoTemper Technologies GmbH). All measurements were performed at RT using hydrophilic capillaries.

### C5b-9 deposition assay

Lpd (5 μg/ml) was immobilized onto microtiter plates (F96 Medisorb, Nunc-Immuno Module) over night at 4°C. Then the plates were washed four times with PBS-Tw and blocked for 1 h at RT with PBS-BSA or blocking buffer I (Applichem). After washings, vitronectin (10–50 μg/ml), clusterin (2.5–20 μg/ml) or Factor H (2.5–50 μg/ml) was added. Following incubation for 1 h at RT the wells were washed, C5b-6 (1.5 μg/ml) and C7 (1 μg/ml) were added and the mixture was incubated for additional 10 min at RT. Thereafter C8 (0.2 μg/ml) and C9 (1 μg/ml) were added. Following incubation for 30 min at 37°C, deposited C5b-9 was detected with a mAb to human C5b-9 (1:1000) and HRP-conjugated swine anti-mouse (1:2500). The reaction was developed with 1,2-phenylenediamine dihydrochloride (OPD, DakoCytomation) and the absorbance was measured at 492 nm.

### Statistics

Results were assessed by Student’s *t*-test for paired data. *p* ≤ 0.05 was considered to be statistically significant. *, *p* ≤ 0.05; **, *p* ≤ 0.01; ***, *p* ≤ 0.001.

## Results

### The Gram-negative bacterium *P*. *aeruginosa* is damaged by host complement

**Fig 1:**
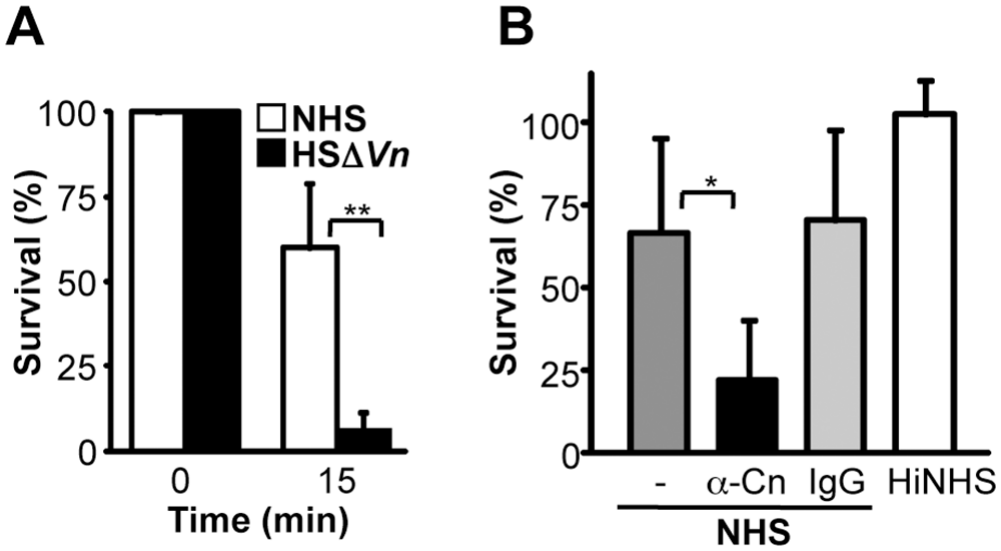
Vitronectin and clusterin bound to *P*. *aeruginosa* protects the bacteria from complement damage. **A**, The relevance of surface bound vitronectin was analyzed by challenging intact bacteria with complement active human serum in which vitronectin was depleted (HSΔ*Vn*). *P*. *aeruginosa* strain SG137 was incubated in HSΔ*Vn* diluted in GVB++ buffer. After incubation, the cells were plated on NB agar plates and the number (CFU) of surviving bacteria was determined. **B**, The relevance of clusterin was analyzed by challenging intact bacteria with complement active human serum in which clusterin activity was blocked. Bacteria were incubated with anti-clusterin mAb or mouse IgG and were thereafter challenged with NHS (1%) diluted in GVB++ buffer. Incubation of bacteria in 1% HiNHS was used as a negative control. Number of bacteria (CFU) at the initiation of all experiments was defined as 100%. The mean values from three independent experiments are shown with error bars indicating SD. *, *p* ≤ 0.05;**, *p* ≤ 0.01.

In order to analyze if *P*. *aeruginosa* influences TCC by exploiting the human TCC regulators vitronectin and clusterin, serum resistance assays were performed. First intact bacteria were challenged with complement active vitronectin depleted human serum (HSΔ*Vn*) (S1 Fig). In the absence of vitronectin, bacteria were more efficiently killed as when challenged with vitronectin containing NHS. In this vitronectin depleted serum bacterial killing was enhanced by 53% (Fig 1A). Thereafter we asked if clusterin, the other human TCC inhibitor contributes to complement control. To this end bacteria were challenge by NHS in which clusterin was blocked by a mAb. Bacterial damage was increased by 44%, when *Pseudomonas* was challenged with NHS in which clusterin function was blocked (Fig 1B). The effect was specific for the clusterin mAb, as an unspecific mouse IgG, which does neither bind to clusterin, nor to *P*. *aeruginosa*, had no effect (data not shown). As bacterial damage is enhanced in the absence of either vitronectin or clusterin, we conclude that each inhibitor is exploited by the pathogen and has protective effect in bacterial survival.

### *P*. *aeruginosa* binds the human terminal pathway regulator vitronectin

**Fig 2:**
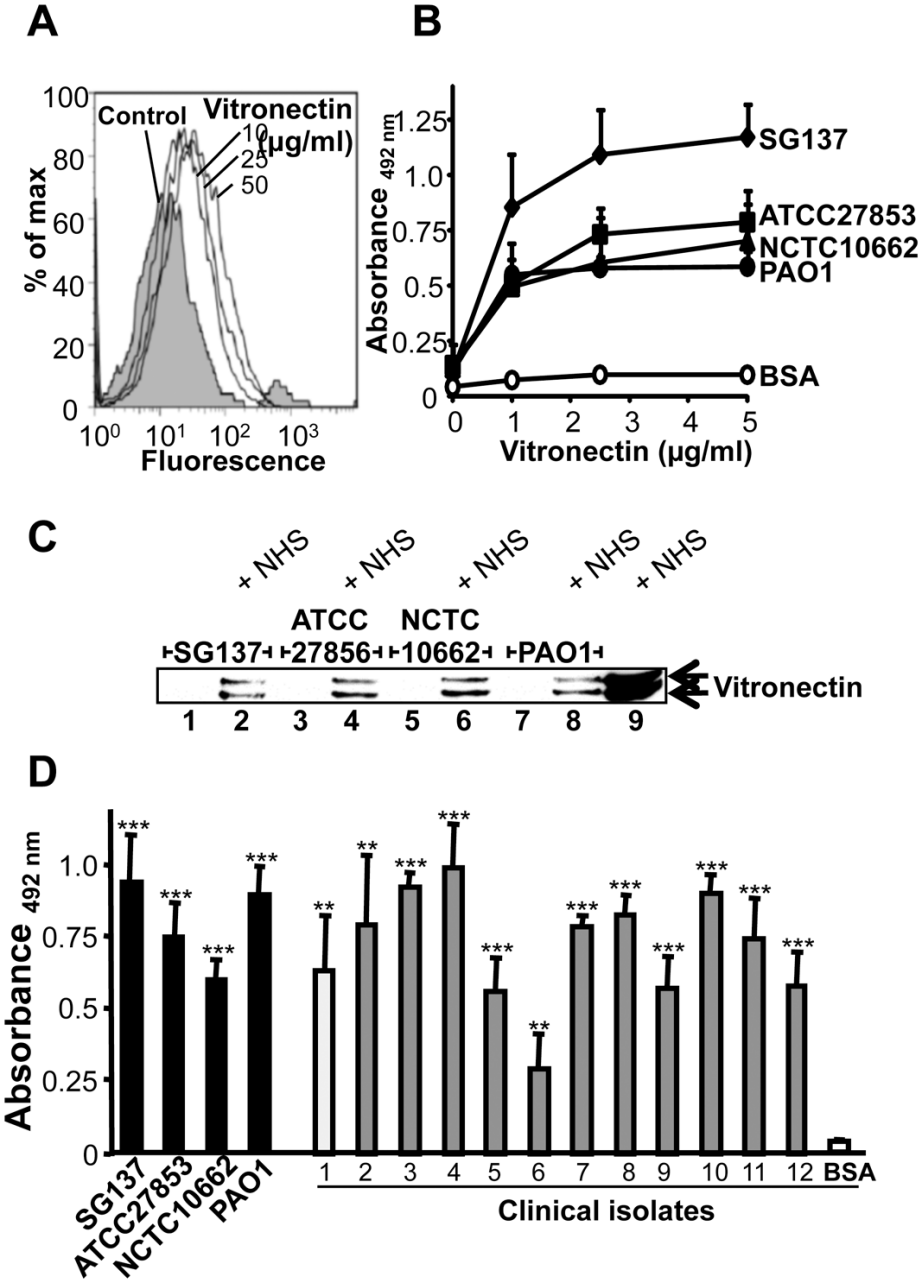
*P*. *aeruginosa* binds the terminal complement regulator vitronectin. **A**, Binding of vitronectin to *P*. *aeruginosa* strain SG137 was assayed by flow cytometry. Bacteria were incubated with vitronectin (10–50 μg/ml) and bound vitronectin was detected with polyclonal vitronectin antiserum and Alexa488-labeled rabbit antiserum. Bacteria incubated with vitronectin specific antiserum and Alexa488-labeled rabbit antiserum served as controls. **B**, Vitronectin binds to *P*. *aeruginosa* and binding was dose-dependent. Four laboratory strains of *P*. *aeruginosa* were analyzed for vitronectin binding using a whole cell ELISA. Whole bacteria were immobilized onto microtiter plates and vitronectin (1–5 μg/ml) was added. Bound vitronectin was detected with polyclonal vitronectin antiserum followed by HRP-conjugated anti-rabbit. **C**, Vitronectin bind to *P*. *aeruginosa*. *P*. *aeruginosa* strains SG137, ATCC 27853, NCTC 10662 and PAO1 were incubated with NHS. Bacteria were washed, lysed, separated by SDS-PAGE and analysed by Western blotting. Bound vitronectin was detected with polyclonal vitronectin antiserum and HRP-conjugated anti-rabbit. A representative experiment of three is shown. **D**, Vitronectin bound to both laboratory and clinical *P*. *aeruginosa* strains. Binding of vitronectin (5 μg/ml) to immobilized bacteria (0.5x10^7^) was assayed by ELISA. Bound vitronectin was detected with polyclonal vitronectin antiserum followed by HRP-conjugated anti-rabbit pAb. The mean values of three independent experiments and standard deviations (SD) are presented. Statistical significance of differences was estimated using Student’s t test. **, *p*≤ 0.01; ***, *p*≤ 0.001.

To define how vitronectin and clusterin contribute to bacterial protection, we next asked if *P*. *aeruginosa* binds these human TCC inhibitors. First binding of purified human vitronectin to *P*. *aeruginosa* strain SG137 was assayed. In this set up, the bacteria were incubated with purified vitronectin and after washing the bound human inhibitor was detected by flow cytometry. Vitronectin bound to the surface of strain SG137 and binding was dose dependent (Fig 2A). Similarly binding of vitronectin to the bacterial strains SG137, ATCC27853, NCTC10662 and PAO1 was assayed in a whole cell ELISA. The bacterial strains were immobilized, vitronectin was bound and after washing, bound vitronectin was detected with a vitronectin specific antiserum in combination with HRP-conjugated anti-rabbit IgG. Purified vitronectin, used at various concentrations bound dose dependently to all four bacterial strains and binding to strain SG137 was of highest intensity (Fig 2B). In addition, binding of serum-derived vitronectin was followed. Bacteria were incubated in NHS, washed, thereafter the cell lysates with bound proteins were separated by SDS-PAGE and vitronectin was identified by Western blot as two bands of 75 and 65 kDa [29]. Serum derived vitronectin bound to the four *P*. *aeruginosa* strains (Fig 2C). In order to assay whether also clinical isolates of *P*. *aeruginosa* bind vitronectin, binding of purified human vitronectin to twelve clinical isolates and to the laboratory *P*. *aeruginosa* strains was analyzed in a whole cell ELISA. Bacteria were immobilized, vitronectin was attached and following washing bound vitronectin was detected with a specific antiserum in combination with HRP-conjugated secondary antibody. Vitronectin bound to each of the tested clinical isolates and to the *P*. *aeruginosa* strains (Fig 2D). Vitronectin bound with different intensity. Vitronectin bound with high intensity to strains SG137, PAO1 and to the clinical isolate #3, #4, and #10 and bound with moderate intensity to strain ATCC 27853 or the remaining clinical isolates. Binding was highest to clinical isolate #4 (i.e. 100%) and varied to 30% (clinical isolate #6).

### *P*. *aeruginosa* binds the human terminal pathway regulator clusterin

**Fig 3:**
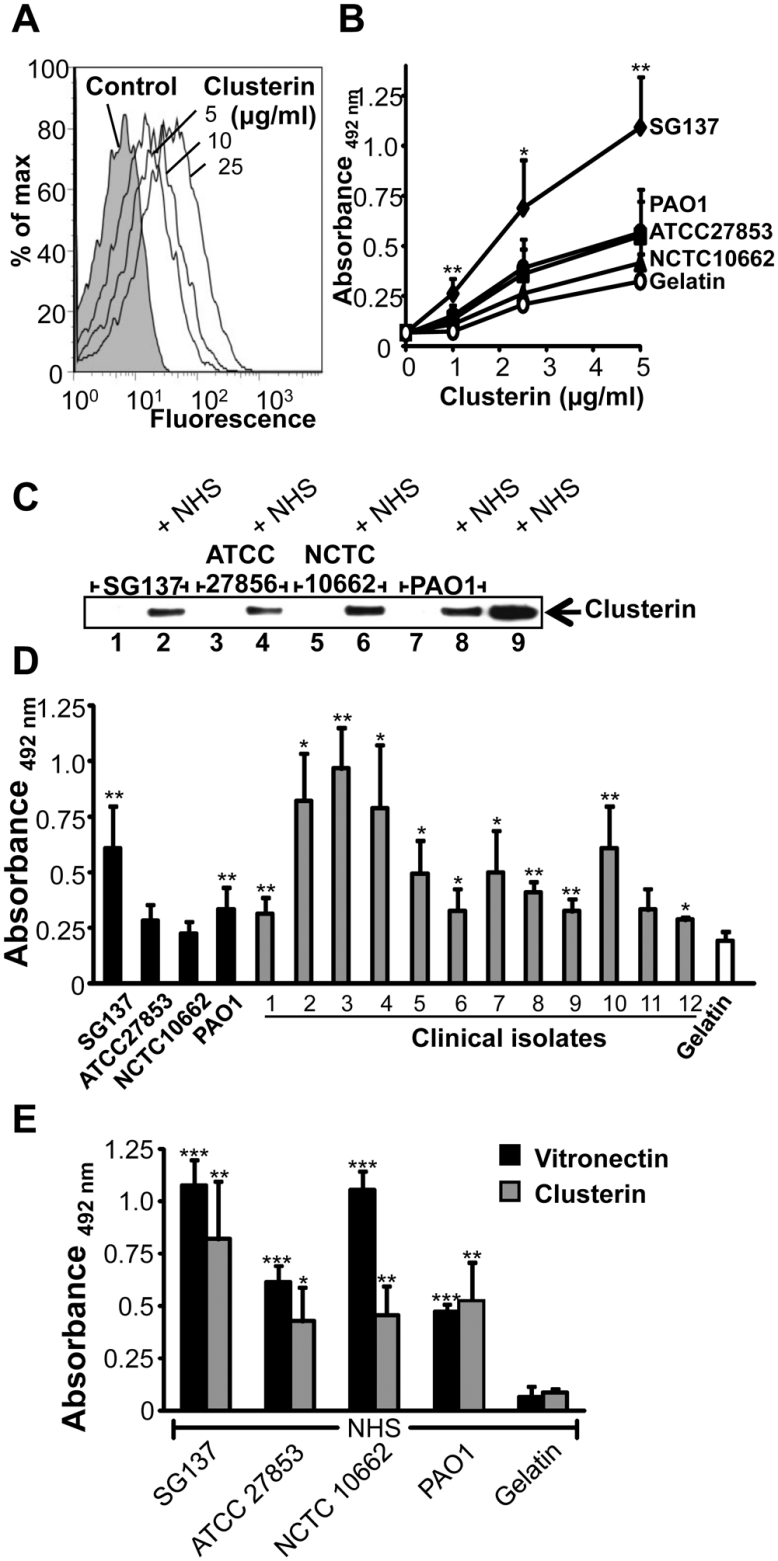
*P*. *aeruginosa* binds the terminal complement regulator clusterin. **A**, Binding of clusterin to *P*. *aeruginosa* strain SG137 was assayed by flow cytometry. Bacteria were incubated with clusterin (5–25 μg/ml) and bound clusterin was detected with a monoclonal clusterin antibody and Alexa488-labeled mouse antiserum. Bacteria incubated with a monoclonal clusterin antibody and Alexa488-labeled mouse antiserum served as controls. **B**, Clusterin binds to intact *P*. *aeruginosa* and binding was dose-dependent. Four laboratory strains of *P*. *aeruginosa* were analyzed for clusterin binding using a whole cell ELISA. Whole bacteria were immobilized onto microtiter plates and clusterin (1–5 μg/ml) was added. Bound clusterin was detected with a monoclonal clusterin antibody followed by HRP-conjugated anti-mouse. **C**, Clusterin bind to *P*. *aeruginosa*. *P*. *aeruginosa* strains SG137, ATCC 27853, NCTC 10662 and PAO1 when incubated with NHS. Bacteria were washed, lysed, separated by SDS-PAGE and analysed by Western blotting. Bound clusterin was detected with a monoclonal clusterin antibody and HRP-conjugated anti-rabbit. A representative experiment of three is shown. **D**, Clusterin bound to both laboratory and clinical *P*. *aeruginosa* strains. Binding of clusterin (2.5 μg/ml) to immobilized bacteria (0.5x10^7^) was assayed by ELISA. Bound clusterin was detected with a monoclonal clusterin antibody followed by HRP-conjugated anti-rabbit pAb. **E**, Binding of vitronectin and clusterin from NHS to immobilized *P*. *aeruginosa* were tested by whole cell ELISA. Bound vitronectin or clusterin was detected with the polyclonal vitronectin antiserum or monoclonal clusterin antibody followed by HRP-conjugated anti-rabbit or mouse pAb. The mean values of three independent experiments and standard deviations (SD) are presented. Statistical significance of differences was estimated using Student’s t test. *, *p*≤ 0.05; **, *p*≤ 0.01, ***, *p*≤ 0.001.

Next binding of the other major terminal pathway regulator clusterin to *P*. *aeruginosa* strain SG137 was assayed. In this set up, the bacteria were incubated with purified clusterin and after washing the bound human inhibitor was detected by flow cytometry. Clusterin bound to the surface of strain SG137 and binding was dose dependent (Fig 3A). Clusterin bound to the *Pseudomonas* strain SG137 in a dose dependent manner (Fig 3B). In addition, binding of serum derived clusterin was followed. Bacteria were incubated in NHS, washed, thereafter the cell lysate with bound proteins were separated by SDS-PAGE and clusterin was identified by Western blotting as a single 80 kDa band. Clusterin purified from serum bound to all four *Pseudomonas* strains (Fig 3C). In addition, clusterin bound with high intensity to strain SG137 and the clinical isolates #2, #3, #4 and #10 and with moderate or low intensity to the other *P*. *aeruginosa* strains # PAO1 and the clinical isolates #1, #5, #6, #7, #8, #9 and #12 (Fig 3D). When adding 2.5 μg/ml of clusterin, the *P*. *aeruginosa* strains ATCC 27853, NCTC 10662 and clinical isolate #11 did not bind. When NHS was used as a source of terminal pathway regulators, both vitronectin and clusterin bound to all four *P*. *aeruginosa* strains (Fig 3E). Serum derived vitronectin bound with the strongest intensity to strain SG137 and to NCTC 10662 and serum derived clusterin to SG137.

### Vitronectin binds to intact *P*. *aeruginosa* via the C-terminal heparin-binding domain (HBD)

**Fig 4:**
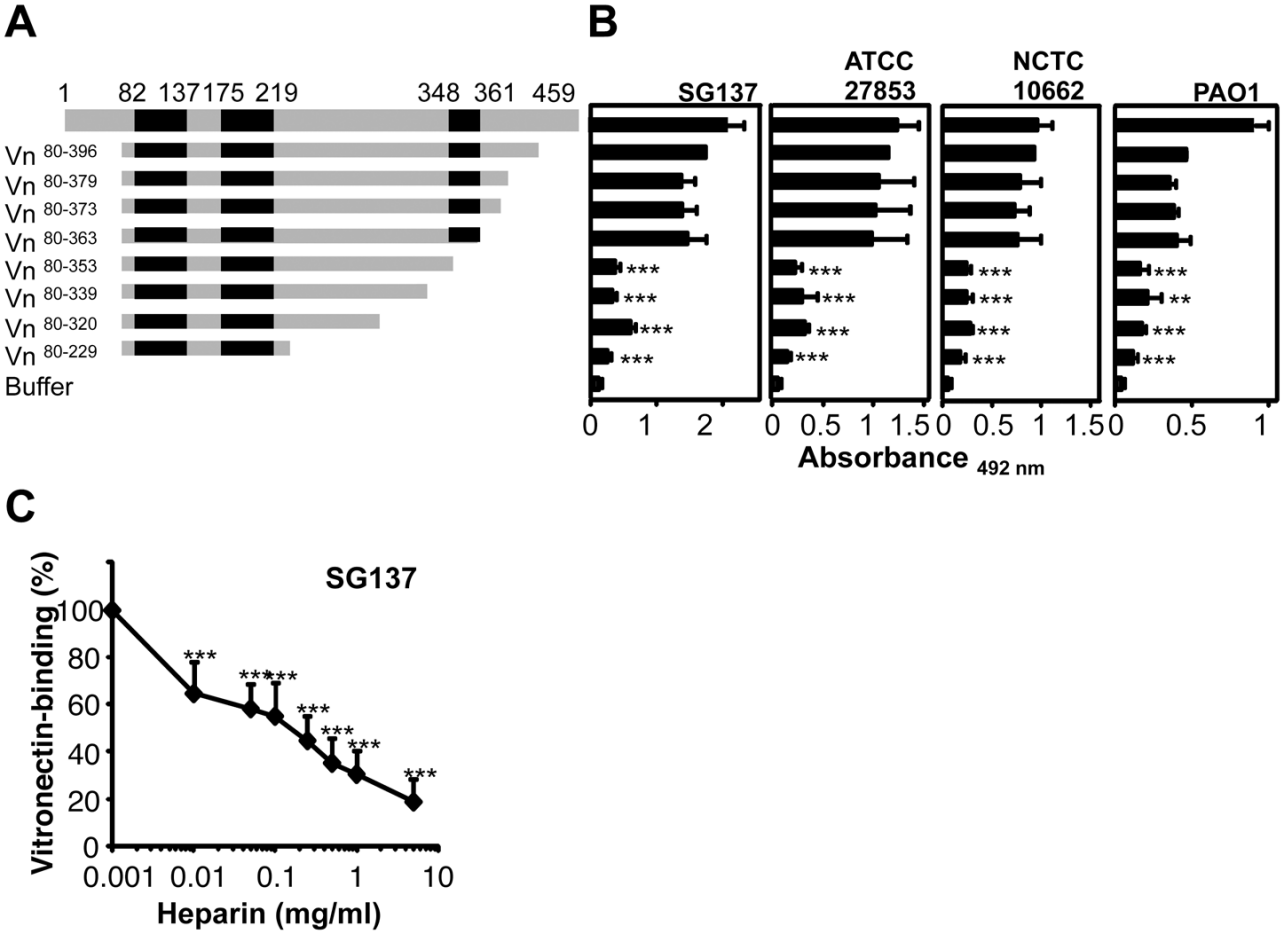
Vitronectin bind to *P*. *aeruginosa* via amino acids 354–363. A, Localization of the region within vitronectin that mediates binding to intact *P*. *aeruginosa*. Vitronectin uses one contact region i.e. aa 354–363 to contact *P*. *aeruginosa*. Vitronectin ^80–396^ (Vn^80-396^) and seven deletion mutants were expressed in HEK cells and purified. The numbers refer to amino acids residues that are included in each construct (*left panel*). Black indicates the heparin binding regions of vitronectin (*left panel*). B, Binding of serum purified full-length vitronectin and vitronectin deletion mutants (5 μg/ml) to immobilized bacteria was assayed by ELISA (*right panel*). Bound vitronectin was detected with polyclonal vitronectin antiserum followed by HRP-conjugated anti-rabbit. **C**, Heparin inhibits binding of vitronectin to *P*. *aeruginosa* strain SG137 and to Lpd, the effect was dose-dependent. The effect of heparin (0.01–5 mg/ml) on vitronectin binding to immobilized *P*. *aeruginosa* strain SG137 was assayed. Bound vitronectin was detected with polyclonal vitronectin antiserum and HRP-conjugated anti-rabbit pAb. The mean values of three independent experiments and SD are presented. Statistical significance of differences was estimated using Student’s t test. **, *p*≤ 0.01; ***, *p*≤ 0.001.

In order to localize the region by which vitronectin attaches to *P*. *aeruginosa*, a series of vitronectin deletion mutants was tested (Fig 4A). Bacteria were immobilized onto microtiter plates, plates were blocked, purified vitronectin and vitronectin fragments were added and vitronectin binding was detected by a vitronectin specific antiserum and HRP-conjugated anti-rabbit. Purified full-length vitronectin and the vitronectin fragment 80–396, which contains all three heparin binding regions, bound to all four *Pseudomonas* strains i.e. SG137, ATCC 27853, NCTC 10662 and PAO1 (Fig 4B). Similarly, the three deletion mutants vitronectin^80-379^, vitronectin^80-373^, vitronectin^80-363^, which include the three HBDs, bound to the *P*. *aeruginosa* strains. In contrast, the deletion mutant vitronectin^80-353^, which lacks eight residues of the third HBD, bound with much lower intensity. Deletion of the 10 amino acids (354–363) reduced binding to the tested *Pseudomonas* strains by 57–88%. Thus, demonstrating that the major contact site for *P*. *aeruginosa* is contained within the C-terminal HBD of vitronectin. Additional deletion mutants, i.e. vitronectin^80-339^ as well as vitronectin^80-229^ bound with similar intensity to *P*. *aeruginosa* as vitronectin^80-353^. Thus, a ten amino acid long region i.e 354–363 contained within the C terminal HBD of vitronectin is relevant for binding to *P*. *aeruginosa*.

Vitronectin has three HBDs to attach heparin. The first HBD is contained within the N-terminal region, spanning from aa 82–137 (HBD I), the second between aa 175–219 (HBD II), and the third (HBD III) is located in the C-terminal part within aa 348–361 [24,46]. We next asked if heparin influences vitronectin binding to *P*. *aeruginosa*. Heparin inhibited vitronectin binding to intact bacteria and the effect was dose-dependent (Fig 4C). Heparin, used at 1 mg/ml reduced vitronectin binding to strain SG137 by 69%. Thus, heparin influences the binding of vitronectin to intact *P*. *aeruginosa*.

### Lpd the *P*. *aeruginosa* protein binds human vitronectin and clusterin

**Fig 5:**
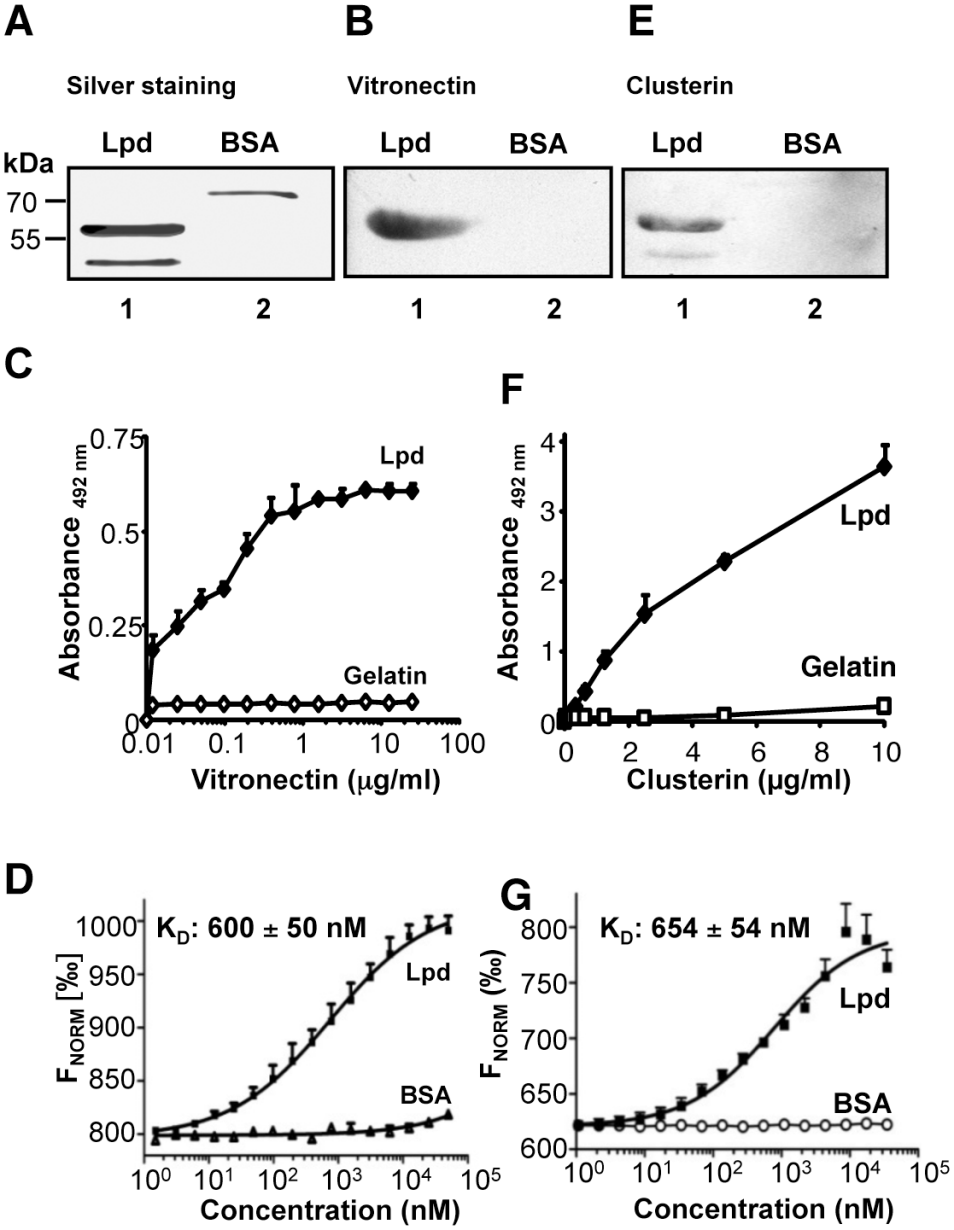
Vitronectin and clusterin bind to recombinant Lpd. **A**, Lpd and BSA were separated by SDS-PAGE and the two proteins were identified by silver staining. The position of the marker proteins is presented on the left. B, Vitronectin binds to Lpd. Lpd and BSA were transferred to a membrane and the membrane was incubated with vitronectin (20 μg/ml). Bound vitronectin was detected with vitronectin specific antiserum and HRP-conjugated anti-rabbit. BSA was used as a negative control. **C**, Vitronectin bound to immobilized Lpd and binding was dose-dependent. Binding of vitronectin (0.01–12.5 μg/ml) to immobilized Lpd was assayed by ELISA. Bound vitronectin was detected with polyclonal vitronectin antiserum and HRP-conjugated anti-rabbit pAb. **D**, Vitronectin binds to Lpd with an affinity of 600 ± 50 nM. Binding of Lpd or BSA used at various concentrations (0.003–50 μM) to NT-647-labeled vitronectin (50 nM) was evaluated in fluid phase by microscale themophoresis (MST). Thermophoresis was recorded at 50% LED power and 80% MST power for 30 s in a Monolith NT.115 instrument. The relative fluorescence in the thermophoresis phase of the experiment was plotted against the concentration of Lpd. **E**, Clusterin binds to Lpd. Lpd and BSA were separated by SDS-PAGE, transferred to a membrane and incubated with vitronectin (20 μg/ml) Bound clusterin was detected with a monoclonal clusterin antibody and HRP-conjugated anti-mouse. BSA was used as a negative control. **F**, Clusterin bound to immobilized Lpd and binding was dose-dependent. Binding of clusterin (0.01–10 μg/ml) to immobilized Lpd was assayed by ELISA. Bound clusterin was detected with anti-clusterin mAb and HRP-conjugated anti-mouse pAb. **G**, Clusterin binds to Lpd with an affinity of 654 ± 54 nM. Binding of Lpd or BSA used at various concentrations (0.001–50 μM) to NT-647-labeled clusterin (25 nM) was evaluated in fluid phase by microscale themophoresis. Thermophoresis was recorded at 50% LED power and 80% MST power for 30 s in a Monolith NT.115 instrument. The relative fluorescence in the thermophoresis phase of the experiment was plotted against the concentration of Lpd.

In order to identify the *Pseudomonas* ligand for human vitronectin and clusterin, we hypothesized that the *Pseudomonas* virulence factor and moonlighting protein Lpd, might serve as a bacterial ligand for both terminal pathway inhibitors [9]. Therefore first binding of vitronectin to Lpd was assayed by ligand affinity blotting. Lpd was separated by SDS-PAGE and either visualized by silver staining or transferred onto a membrane, then vitronectin was added and after washing, the bound regulator was detected. Silver staining identified two Lpd bands, one of 57 and one of 50 kDa (Fig 5A). Vitronectin bound to the 57 kDa Lpd protein (Fig 5B). In addition, binding of human vitronectin to immobilized Lpd was evaluated by ELISA. Vitronectin bound to immobilized Lpd, binding was dose-dependent and saturation was reached at 6.25 μg/ml (Fig 5C). The affinity of the vitronectin/Lpd interaction was determined by microscale thermophoresis. Fluorescently labeled vitronectin was added to Lpd, which was used at serial dilutions and the interaction was followed by MST. Vitronectin bound Lpd with an affinity of 600 ± 50 nM (Fig 5D). Thus, *Pseudomonas* Lpd is a bacterial ligand for the humn TCC inhibitor vitronectin.

In addition, we tested whether Lpd also binds clusterin. Again Lpd was separated by SDS-PAGE, transferred onto a membrane, then clusterin was added and after washing, the bound regulator was detected with the specific mAb. Clusterin bound to the 57 kDa Lpd protein (Fig 5E). In addition, clusterin bound to Lpd in an ELISA assay and binding was dose-dependent (Fig 5F). Clusterin bound Lpd with an affinity of 654 ± 54 nM, as shown by MST (Fig 5G). Thus, Lpd is a surface ligand of *Pseudomonas* that binds the two human terminal pathway inhibitors.

### The C-terminal HBD of vitronectin (i.e. 354–396) is the major binding site for Lpd

**Fig 6:**
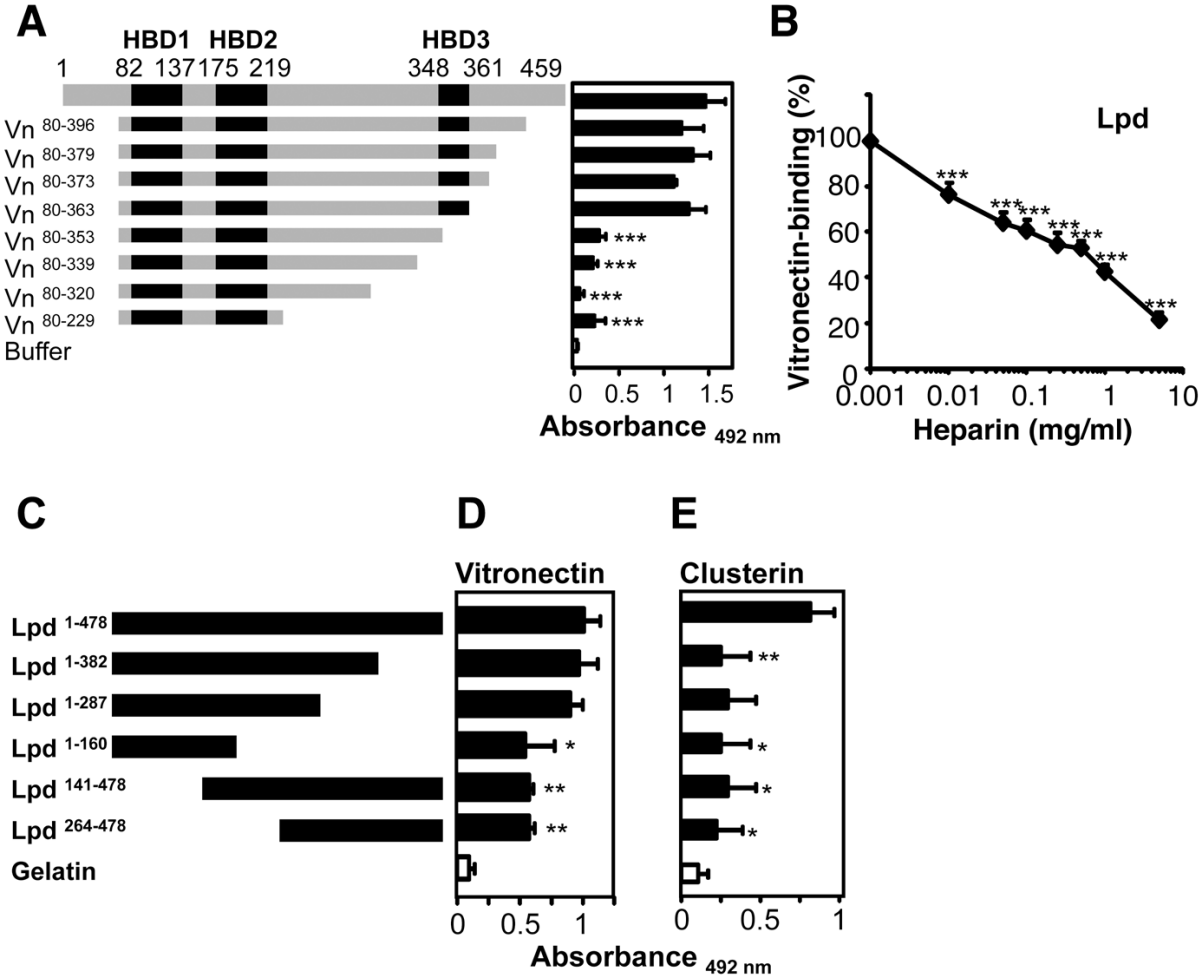
Localization of binding regions. **A**, Localization of the region within vitronectin that mediates binding to Lpd. Vitronectin uses one contact region i.e. aa 354–363 to contact Lpd. Vitronectin ^80–396^ (Vn^80-396^) and seven deletion mutants were expressed in HEK cells and purified. The numbers refer to amino acids residues that are included in each construct (*left panel*). Black indicates the heparin binding regions of Vitronectin (*left panel*). Binding of serum purified full-length vitronectin and vitronectin deletion mutants (5 μg/ml) to immobilized Lpd was assayed by ELISA (*right panel*). Bound vitronectin was detected with polyclonal vitronectin antiserum followed by HRP-conjugated anti-rabbit. **B**, Heparin inhibits binding of vitronectin to Lpd and the effect was dose-dependent. The effect of heparin (0.01–5 mg/ml) on vitronectin binding to immobilized Lpd was assayed. Bound vitronectin was detected with polyclonal vitronectin antiserum and HRP-conjugated anti-rabbit pAb. **C**, Schematic picture of full length Lpd and its fragments. **D**, The vitronectin-binding regions are located within two separate binding domains of Lpd. Equimolar amounts (13.3 nM) of full length Lpd and Lpd deletion mutants were immobilized onto microtiter plates, vitronectin was added and bound vitronectin was quantified. **E**, The clusterin-binding regions are located within two separate binding domains of Lpd. Equimolar amounts (13.3 nM) of full length Lpd and Lpd deletion mutants were immobilized onto microtiter plates, clusterin was added and bound clusterin was quantified. The mean values of three independent experiments and SD are presented. Statistical significance of differences was estimated using Student’s t test. *, *p*≤ 0.05; **, *p*≤ 0.01.

In order to localize the binding regions of vitronectin for bacterial Lpd, binding of serum purified full-length vitronectin and the various vitronectin deletion mutants to immobilized Lpd was tested. Full-length vitronectin and each vitronectin deletion mutant, which includes the third, C terminal HBD, i.e. vitronectin ^80–396^, vitronectin ^80–379^, vitronectin ^80–373^, vitronectin ^80–363^ bound to immobilized Lpd with high intensity (Fig 6A). Deletion mutant vitronectin ^80–353^, which has 10 additional C terminal residues deleted and which lacks the C terminal HBD, bound with lower intensity (Δ70%). The additional deletion mutants, i.e. vitronectin^80-339^ and vitronectin^80-229^ also bound with rather low intensity. Thus, indicating that the 10 residues, located within the C terminal heparin binding region, i.e. amino acids 354–363 of vitronectin represent the major binding region for *Pseudomonas* Lpd. As the C-terminal HBD III of vitronectin contributes to Lpd binding, we tested how heparin influences the vitronectin/Lpd interaction. Heparin inhibited vitronectin binding to Lpd, and used at 1 mg/ml, reduced vitronectin binding to Lpd by 57% (Fig 6B). Thus, heparin influences binding of vitronectin to purified Lpd and to intact *P*. *aeruginosa* in a similar manner. This shows that Lpd is a surface ligand of *Pseudomonas* for the two human terminal pathway inhibitors.

### Lpd has two binding sites for human vitronectin

In order to localize the binding sites within the bacterial Lpd protein for both human TCC regulators, five Lpd deletion mutants were generated, i.e. Lpd^1-382^: 53 kDa, Lpd^1-287^: 37 kDa; Lpd^1-160^: 20 kDa, Lpd^141-478^: 42 kDa and Lpd^264-478^: 27 kDa, expressed and purified (S2 Fig and Fig 6C). First binding of vitronectin to the immobilized deletion mutants was assayed by ELISA. Vitronectin bound to full-length Lpd (Lpd^1-478^) and to the fragments containing the N-terminus and middle region (i.e. Lpd^1-382^ and Lpd^1-287^) and binding was of similar intensity (Fig 6D). Deletion of the middle region (aa 161–287) of Lpd protein reduced vitronectin binding by 51%. However, also deletion of the N-terminal residues 1–141 (Lpd^141-478^) and 1–263 (Lpd^264-^478) reduced the binding by 47%. Thus, suggesting that Lpd uses two regions to bind human vitronectin. One interaction region is located in the middle region (aa 161–287) and the second region in the C-terminus (aa 264–478) of Lpd.

### Lpd has two binding sites for human clusterin

Similar, also clusterin bound to full length Lpd^1-478^ (Fig 6E). Clusterin binding to the Lpd deletion mutants was reduced by 64–70%. When the C-terminus and middle region of Lpd was deleted, clusterin bound to the N-terminal Lpd^1-160^ with about 70% lower intensity. As deletion of the C-terminus (i.e. residues 383 to 478) reduced binding, one major binding site is contained within the C terminal region. In addition, as clusterin bound to all other deletion mutants with low intensity one additional binding site is located in the N terminal segment between residues 1 to 160. Thus, suggesting that *Pseudomonas* Lpd uses two regions to bind human clusterin. One interaction region is included within the N-terminusa (i.e. aa 1–160) and the second region within the C-terminus (aa 382–478) of Lpd.

### Human complement regulators bind to Lpd simultaneously

**Fig 7:**
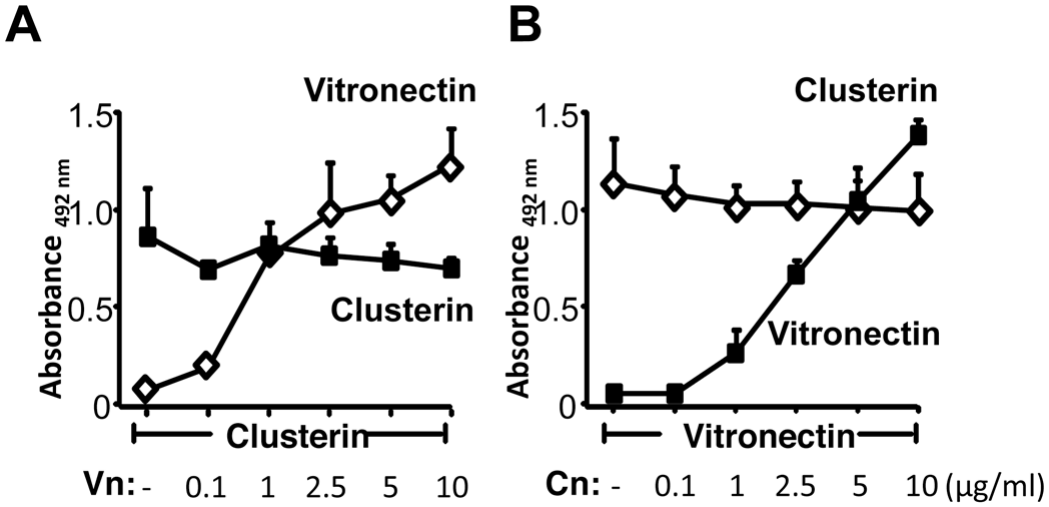
Vitronectin and clusterin bind simultaneously to Lpd. **A,** Effect of increasing clusterin levels in the presence of a constant concentration of vitronectin. Binding of clusterin (used at the indicated concentrations) and vitronectin (5 μg/ml) to immobilized Lpd was analysed by ELISA. Bound clusterin was detected with anti-clusterin mAb (■) and bound vitronectin was detected with anti-vitronectin mAb (◇). **B,** In a reverse setting, the clusterin concentration was kept constant (2.5 μg/ml) and binding of vitronectin (used at the indicated concentrations) was evaluated. The mean values of three independent experiments and SD are presented.

*Pseudomonas* Lpd is a bacterial moonlighting protein that binds vitronectin and clusterin and also the additional human complement regulators, Factor H and plasminogen. Factor H and plasminogen bind to distinct regions in the Lpd protein [9]. Given this large repertoire of human regulators which all bind to *Pseudomonas* Lpd we next asked if vitronectin and clusterin bind independently form each other or if they compete with each other for binding. First we analyzed if vitronectin affects clusterin binding to Lpd. In the presence of clusterin, vitronectin when used at increasing concentrations bound in a dose-dependent manner to immobilized Lpd and did not influence clusterin binding (Fig 7A). Similarly, clusterin when used at increasing concentrations bound dose depently to immobilized Lpd and did not affect vitronectin binding (Fig 7B).

Similarly, we assayed if vitronectin influences Factor H binding to Lpd. Vitronectin did influence Factor H binding (S3A Fig). Vitronectin used at four-fold molar excess reduced Factor H binding by 37% (S3A Fig). In a reverse setting, Factor H used at increasing concentrations did not influence vitronectin binding (S3B Fig). At physiological levels, e.g. at a molar ratio 0.5:1 (vitronectin:Factor H), both human complement regulators bound to Lpd simultaneously. Thus, several different human plasma proteins apparently bind simultaneously and independent from each other to bacterial Lpd.

### Vitronectin and plasminogen bind to Lpd simultaneously

In addition, we asked if vitronectin and plasminogen bind simultaneously to Lpd or if the two human proteins compete with each other for Lpd binding. Vitronectin in the presence of plasminogen bound to Lpd and binding was dose dependent (S3C Fig). Vitronectin used at increasing levels did not influence plasminogen binding. Similarly, plasminogen bound dose dependently to Lpd and plasminogen did not influence vitronectin binding (S3D Fig). Thus, plasminogen and vitronectin bind to separate sites in Lpd and the human complement regulators do not compete for binding.

### Vitronectin and clusterin bound to Lpd inhibits the terminal complement pathway

**Fig 8:**
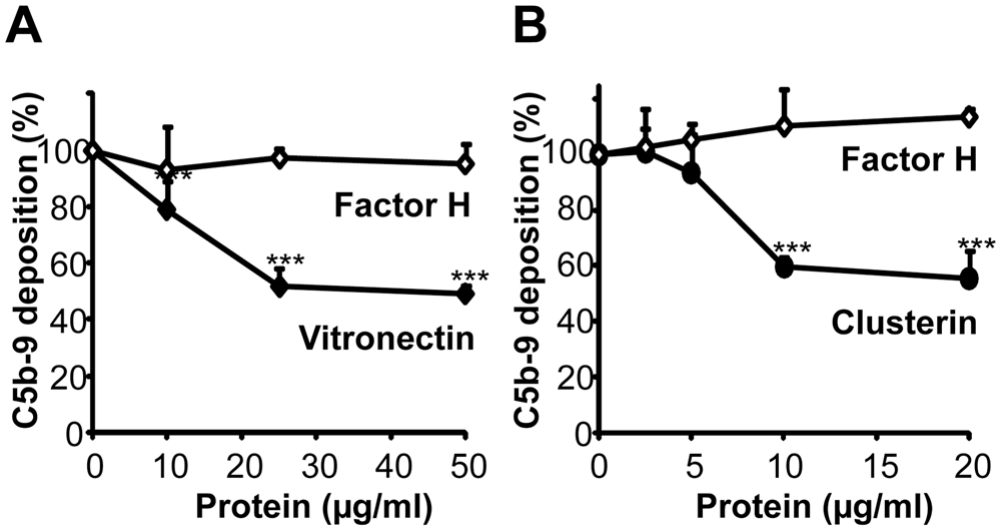
The terminal pathway regulators vitronectin and clusterin when bound to Lpd are functionally active. Both vitronectin (**A**) and clusterin (**B**) when bound to immobilized Lpd inhibits C5b-9 deposition. **A**, Vitronectin (10–50 μg/ml) or Factor H (10–50 μg/ml) was bound to immobilized Lpd and after extensive washing C5b-6 and C7 were added. After 10 min incubation C8 and C9 were added and C5b-9 deposition was detected with mouse anti-C5b-9 mAb and HRP-conjugated anti-mouse pAb. **B**, Clusterin (2.5–20 μg/ml) or Factor H (2.5–20 μg/ml) was bound to immobilized Lpd and after extensive washing C5b-6 and C7 were added. After 10 min incubation C8 and C9 were added and C5b-9 deposition was detected with mouse anti-C5b-9 mAb and HRP-conjugated anti-mouse pAb. The mean values of three independent experiments and SD are presented. Statistical significance of differences was estimated using Student’s t test. ***, *p*≤ 0.001.

Given that the two human TCC inhibitors bind to Lpd and to the bacterial surface we asked if the attached human inhibitors are functionally active. To this end we evaluated if vitronectin or clusterin bound to *Pseudomonas* Lpd are functionally active and inhibit TCC formation. To this end first vitronectin or clusterin was bound to Lpd, then the terminal components C5b-6 and C7 were added and after 10 min C8 and C9 were added. Following incubation for 30 min, C5b-9 (TCC) deposition was followed. Vitronectin when bound to Lpd, blocked C5b-9 deposition and the effect was dose dependent (Fig 8A). Vitronectin used at 25 μg/ml (i.e. below the plasma levels of 200–700 μg/ml) inhibited C5b-9 deposition by 48%. Similar, clusterin used at 20 μg/ml (i.e. below plasma levels of 35–135 μg/ml) inhibited C5b-9 deposition by 45% (Fig 8B). Thus, both human TCC regulators when bound to Lpd blocked TCC formation and deposition.

## Discussion

Here we identify Lpd as the first vitronectin and clusterin binding protein of the Gram-negative bacterium *P*. *aeruginosa*. Lpd is exposed at the bacterial surface and binds the human terminal pathway inhibitors vitronectin and clusterin. Vitronectin as well as clusterin bound to Lpd are functionally active and block complement TCC formation and thereby contribute to bacterial innate immune escape.

Lpd is the first vitronectin and clusterin binding protein identified from the human pathogenic bacterium *P*. *aeruginosa*. Both human terminal pathway regulators bound to the majority of the tested *P*. *aeruginosa* laboratory strains and clinical isolates. Lpd is a moonlighting protein that is exposed on the bacterial surface and is also present in the cytoplasm [9,11,12]. Lpd exposed at the bacterial surface binds several human complement regulators and thereby contributes to innate immune escape. In addition to bind vitronectin and clusterin, Lpd also attaches Factor H, FHL-1, CFHR1, as well as plasminogen [9]. Both vitronectin and clusterin bind Lpd with a similar affinity of ∼600 nM. Bound to Lpd, each human regulator maintains its regulatory function and blocks TCC assembly and formation. Vitronectin uses a region of ten residues (i.e. amino acids 354–363) to attach to intact *P*. *aeruginosa* and also to purified Lpd. This region is contained within the third, C-terminal HBD of vitronectin (i.e. 348–361) [22,24,46]. This explains why heparin inhibits vitronectin binding to both intact bacteria and to purified Lpd. By attaching to Lpd via the C-terminal amino acid residues 354–363 vitronectin is orientated in such manner that Lpd attached vitronectin exposes its functional N-terminal region, i.e. aa 51–310 [47]. Thus, Lpd attached vitronectin can therefore bind to the metastable binding site of the C5b-7 complex and block membrane insertion of the C5b-7 complex and consequentially terminal pathway [47].

Lpd exposed on the bacterial surface of *P*. *aeruginosa* binds three human terminal pathway inhibitors i.e. vitronectin, clusterin and CFHR1 and also the three C3 convertase inhibitors Factor H and FHL-1 and plasminogen [9]. Apparently the human regulators vitronectin, clusterin, Factor H and plasminogen bind to separate sites in the bacterial Lpd protein. When attached to Lpd, each inhibitor is functionally active and blocks complement action and the generation of effector components. Thus, by attaching a series of human complement regulators, *P*. *aeruginosa* controls and modulates host innate immune response rather efficiently and can influence complement cascade progression at multiple levels.

Acquisition of vitronectin from human serum is a rather common evasion strategy that is used by many pathogenic microbes. Similar to *P*. *aeruginosa*, other Gram-negative bacteria e.g. *H*. *influenzae*, *M*. *catarrhalis* and *N*. *meningitidis*, as well as the Gram-positive bacterium *S*. *pneumoniae* and the pathogenic fungus *C*. *albicans* bind human vitronectin [33,34,48–51]. Each of these microbes utilizes surface attached vitronectin for complement escape, for interaction with human cells and/or for ECM attachment. Several microbial vitronectin binding proteins are identified. In addition to Lpd of *P*. *aeruginosa*, the other microbial vitronectin ligands include Protein E (*H*. *influenzae*), PspC (*S*. *pneumoniae*) and Gpm1 (*C*. *albicans*) [36,50,51]. Several pathogenic microbes and their corresponding binding proteins attach to vitronectin via the third heparin binding region and apparently the same C-terminal 10 amino acid long segment represents the major binding region of vitronectin. This related pattern explains why in all cases heparin reduce vitronectin binding to each of these microbial proteins and that the inhibitory effects are comparable 50–85% [36,50,51]. Thus, four immune evasion proteins derived from Gram-negative bacteria, a Gram-positive bacterium or from a fungal pathogen all attach vitronectin via the same region. This use of an identical binding site in the human TCC inhibitor, combined with the related binding characteristics, identify common patterns for regulator attachment and for microbial immune evasion. This reveals a conserved strategy for vitronectin recruitment.

At present binding of clusterin to the various human pathogenic microbes is not understood in such a detail. So far a limited number of microbial pathogens have been identified which bind this regulator. These include *S*. *aureus*, *S*. *epidermidis* and *S*. *pyogenes* and at present one single bacterial protein, i.e. SIC from *S*. *pyogenes* was identified as a clusterin ligand [42,44,45]. Binding of clusterin contributes to complement inhibition and to bacterial aggregation. Furthermore, the interaction between clusterin and dengue virus is suggested to contribute to pathogenesis of the virus, as the virus interfere with the complement inhibitory functions of clusterin [43].

In summary, both *P*. *aeruginosa* laboratory strains and clinical isolates bound the terminal pathway regulator vitronectin and bound to the surface of the bacterium, vitronectin protected from complement attack. When challenged with complement active human serum depleted from vitronectin the bacterium was severely damaged and bacterial survival was reduced by over 50%. Similarly, *P*. *aeruginosa* also bound to the other major terminal pathway regulator clusterin. When clusterin in human serum was blocked by a mAb, bacterial survival was reduced by 44%. Thus, the capacity to bind terminal pathway inhibitors contributes to survival of this Gram-negative bacterium in human serum. In the present study we identified Lpd as the first immune evasion protein of the Gram-negative bacterium *P*. *aeruginosa* that binds both vitronectin and clusterin. Lpd is a multifunctional virulence factor that in addition to vitronectin and clusterin binds the terminal pathway inhibitor CFHR1 and three additional human complement regulators that control the human complement at the level of the C3 convertase. Simultaneous binding of several human complement inhibitors by one surface exposed bacterial protein, allows the pathogen to block the complement attack at multiple levels, including blockade of the C3 convertase and of the terminal complement pathway.

## Supporting Information

S1 Fig Depletion of vitronectin from NHS. A, Human serum was depleted from vitronectin by affinity chromatography using rabbit anti-human vitronectin. NHS was incubated with the antibody-coated Sepharose, followed by centrifugation to collect the depleted serum. The vitronectin-depleted serum (HSΔ*Vn*) was analyzed for the presence of vitronectin by Western blotting using polyclonal vitronectin antiserum. B, Densitometry measurements revealed a 80% depletion of vitronectin. The mean values of three independent experiments and SD are presented. Statistical significance of differences was estimated using Student’s t test. ***, *p*≤ 0.001.
(PPTX)

S2 Fig Recombinant expression of *P*. *aeruginosa* Lpd deletion mutants. Recombinant full length Lpd and Lpd deletion mutants were expressed as a N-terminal His-tag protein. The Lpd encoding cDNA was amplified from genomic DNA derived from *P*. *aeruginosa* strain PAO1 by PCR, cloned into expression vector pET200D and the corresponding protein was recombinantly expressed in *E*. *coli* with a N-terminal His-tag. Recombinant Lpd deletion mutants were purified by affinity chromatography. The purified proteins were separated by SDS-PAGE and analyzed by silver staining. The mobility of the size markers are indicated on the left in kDa.
(PPTX)

S3 Fig Vitronectin, Factor H and plasminogen bind simultaneously to Lpd. A, Effect of increasing vitronectin levels in the presence of a constant concentration of Factor H (molar ratios are shown). Binding of vitronectin (used at the indicated concentrations) and Factor H (5 μg/ml) to immobilized Lpd was analysed by ELISA. Bound vitronectin was detected with vitronectin antiserum (■) and bound Factor H was detected with Factor H antiserum(◇). B, In a reverse setting, the vitronectin concentration was kept constant (5 μg/ml) and binding of Factor H (used at the indicated concentrations) was evaluated (molar ratios are shown). C, Effect of increasing vitronectin levels in the presence of a constant concentration of plasminogen. Binding of vitronectin (used at the indicated concentrations) and plasminogen (5 μg/ml) to immobilized Lpd was analysed by ELISA (molar ratios are shown). Bound vitronectin was detected with vitronectin antiserum (■) and bound plasminogen was detected with plasminogen antiserum (◇). D, In a reverse setting, the vitronectin concentration was kept constant (5 μg/ml) and binding of plasminogen (used at the indicated concentrations) was evaluated (molar ratios are shown). The mean values of three independent experiments and SD are presented. Statistical significance of differences was estimated using Student’s t test. *, *p*≤ 0.05.
(PPTX)

## References

[1] Pier GB, Ramphal R. *Pseudomonas aeruginosa*. 6th ed New York: Churchill Livingstone; 2005.

[2] Hassett DJ, Korfhagen TR, Irvin RT, Schurr MJ, Sauer K, Lau GW, et al *Pseudomonas aeruginosa* biofilm infections in cystic fibrosis: insights into pathogenic processes and treatment strategies. Expert Opin Ther Targets. 2010;14: 117–130. 10.1517/14728220903454988 20055712

[3] Sadikot RT, Blackwell TS, Christman JW, Prince AS. Pathogen-host interactions in *Pseudomonas aeruginosa* pneumonia. Am J Respir Crit Care Med. 2005;171: 1209–1223. 15695491 10.1164/rccm.200408-1044SO PMC2718459

[4] Fujitani S, Sun H-Y, Yu VL, Weingarten JA. Pneumonia due to *Pseudomonas aeruginosa*: part I: epidemiology, clinical diagnosis, and source. Chest. 2011;139: 909–919. 10.1378/chest.10-0166 21467058

[5] Chastre J, Fagon J-Y. Ventilator-associated pneumonia. Am J Respir Crit Care Med. 2002;165: 867–903. 10.1164/ajrccm.165.7.2105078 11934711

[6] Laarman AJ, Bardoel BW, Ruyken M, Fernie J, Milder FJ, van Strijp JAG, et al *Pseudomonas aeruginosa* alkaline protease blocks complement activation via the classical and lectin pathways. J Immunol. 2012;188: 386–393. 10.4049/jimmunol.1102162 22131330

[7] Schiller NL, Joiner KA. Interaction of complement with serum-sensitive and serum-resistant strains of *Pseudomonas aeruginosa* . Infect Immun. 1986;54: 689–694. 3096887 10.1128/iai.54.3.689-694.1986 PMC260224

[8] Engels W, Endert J, Van Boven CP. A quantitative method for assessing the third complement factor (C3) attached to the surface of opsonized *Pseudomonas aeruginosa*: interrelationship between C3 fixation, phagocytosis and complement consumption. J Immunol Methods. 1985;81: 43–53. 3926902 10.1016/0022-1759(85)90120-6

[9] Hallström T, Mörgelin M, Barthel D, Raguse M, Kunert A, Hoffmann R, et al Dihydrolipoamide dehydrogenase of *Pseudomonas aeruginosa* is a surface-exposed immune evasion protein that binds three members of the factor H family and plasminogen. J Immunol. 2012;189: 4939–4950. 10.4049/jimmunol.1200386 23071278

[10] Hong YQ, Ghebrehiwet B. Effect of *Pseudomonas aeruginosa* elastase and alkaline protease on serum complement and isolated components C1q and C3. Clin Immunol Immunopathol. 1992;62: 133–138. 1730152 10.1016/0090-1229(92)90065-v

[11] McCully V, Burns G, Sokatch JR. Resolution of branched-chain oxo acid dehydrogenase complex of *Pseudomonas aeruginosa* PAO. Biochem J. 1986;233: 737–742. 3085653 10.1042/bj2330737 PMC1153093

[12] Stover CK, Pham XQ, Erwin AL, Mizoguchi SD, Warrener P, Hickey MJ, et al Complete genome sequence of *Pseudomonas aeruginosa* PAO1, an opportunistic pathogen. Nature. 2000;406: 959–964. 10.1038/35023079 10984043

[13] Zipfel PF, Hallström T, Riesbeck K. Human complement control and complement evasion by pathogenic microbes—tipping the balance. Mol Immunol. 2013;56: 152–160. 10.1016/j.molimm.2013.05.222 23810413

[14] Walport MJ. Complement. First of two parts. N Engl J Med. 2001;344: 1058–1066. 11287977 10.1056/NEJM200104053441406

[15] Nishida N, Walz T, Springer TA. Structural transitions of complement component C3 and its activation products. Proc Natl Acad Sci. 2006;103: 19737–19742. 10.1073/pnas.0609791104 17172439 PMC1750921

[16] Ricklin D, Hajishengallis G, Yang K, Lambris JD. Complement: a key system for immune surveillance and homeostasis. Nat Immunol. 2010;11: 785–797. 10.1038/ni.1923 20720586 PMC2924908

[17] Bubeck D. The making of a macromolecular machine: assembly of the membrane attack complex. Biochem. 2014;53: 1908–1915. 10.1021/bi500157z 24597946

[18] Hadders MA, Bubeck D, Roversi P, Hakobyan S, Forneris F, Morgan BP, et al Assembly and regulation of the membrane attack complex based on structures of C5b6 and sC5b9. Cell Rep. 2012;1: 200–207. 10.1016/j.celrep.2012.02.003 22832194 PMC3314296

[19] Liszewski M, Atkinson JP. Complement regulators in human disease: lessons from modern genetics. J Intern Med. 2015;277: 294–305. 10.1111/joim.12338 25495259

[20] Zipfel PF, Skerka C. Complement regulators and inhibitory proteins. Nat Rev Immunol. 2009;9: 729–740. 10.1038/nri2620 19730437

[21] Kunert A, Losse J, Gruszin C, Hühn M, Kaendler K, Mikkat S, et al Immune evasion of the human pathogen Pseudomonas aeruginosa: elongation factor Tuf is a factor H and plasminogen binding protein. J Immunol Baltim Md 1950. 2007;179: 2979–2988. 10.4049/jimmunol.179.5.2979 17709513

[22] Singh B, Su Y-C, Riesbeck K. Vitronectin in bacterial pathogenesis: a host protein used in complement escape and cellular invasion. Mol Microbiol. 2010;78: 545–560. 10.1111/j.1365-2958.2010.07373.x 20807208

[23] Podack ER, Preissner KT, Müller-Eberhard HJ. Inhibition of C9 polymerization within the SC5b-9 complex of complement by S-protein. Acta Pathol Microbiol Immunol Scand Suppl. 1984;284: 89–96. 6587746

[24] Preissner KT, Jenne D. Structure of vitronectin and its biological role in haemostasis. Thromb Haemost. 1991;66: 123–132. 1718050

[25] Preissner KT, Podack ER, Müller-Eberhard HJ. The membrane attack complex of complement: relation of C7 to the metastable membrane binding site of the intermediate complex C5b-7. J Immunol. 1985;135: 445–451. 3998468

[26] Guo W, Ma X, Xue C, Luo J, Zhu X, Xiang J, et al Serum clusterin as a tumor marker and prognostic factor for patients with esophageal cancer. Dis Markers. 2014;2014: 168960 10.1155/2014/168960 25574066 PMC4276701

[27] Choi NH, Mazda T, Tomita M. A serum protein SP40,40 modulates the formation of membrane attack complex of complement on erythrocytes. Mol Immunol. 1989;26: 835–840. 2601725 10.1016/0161-5890(89)90139-9

[28] Jenne DE, Tschopp J. Molecular structure and functional characterization of a human complement cytolysis inhibitor found in blood and seminal plasma: identity to sulfated glycoprotein 2, a constituent of rat testis fluid. Proc Natl Acad Sci. 1989;86: 7123–7127. 2780565 10.1073/pnas.86.18.7123 PMC298007

[29] Schvartz I, Seger D, Shaltiel S. Vitronectin. Int J Biochem Cell Biol. 1999;31: 539–544. 10399314 10.1016/s1357-2725(99)00005-9

[30] Millis AJ, Luciani M, McCue HM, Rosenberg ME, Moulson CL. Clusterin regulates vascular smooth muscle cell nodule formation and migration. J Cell Physiol. 2001;186: 210–219. 10.1002/1097-4652(200102)186:2<210::AID-JCP1019>3.0.CO;2-N 11169458

[31] Silkensen JR, Skubitz KM, Skubitz AP, Chmielewski DH, Manivel JC, Dvergsten JA, et al Clusterin promotes the aggregation and adhesion of renal porcine epithelial cells. J Clin Invest. 1995;96: 2646–2653. 10.1172/JCI118330 8675630 PMC185970

[32] Arko RJ, Chen CY, Schalla WO, Sarafian SK, Taylor CL, Knapp JS, et al Binding of S protein by *Neisseria gonorrhoeae* and potential role in invasion. J Clin Microbiol. 1991;29: 70–75. 1704384 10.1128/jcm.29.1.70-75.1991 PMC269705

[33] Attia AS, Ram S, Rice PA, Hansen EJ. Binding of vitronectin by the *Moraxella catarrhalis* UspA2 protein interferes with late stages of the complement cascade. Infect Immun. 2006;74: 1597–1611. 16495531 10.1128/IAI.74.3.1597-1611.2006 PMC1418666

[34] Griffiths NJ, Hill DJ, Borodina E, Sessions RB, Devos NI, Feron CM, et al Meningococcal surface fibril (Msf) binds to activated vitronectin and inhibits the terminal complement pathway to increase serum resistance. Mol Microbiol. 2011;82: 1129–1149. 10.1111/j.1365-2958.2011.07876.x 22050461

[35] Leroy-Dudal J, Gagnière H, Cossard E, Carreiras F, Di Martino P. Role of alphavbeta5 integrins and vitronectin in *Pseudomonas aeruginosa* PAK interaction with A549 respiratory cells. Microbes Infect Inst Pasteur. 2004;6: 875–881. 10.1016/j.micinf.2004.05.004 15310463

[36] Singh B, Jalalvand F, Mörgelin M, Zipfel P, Blom AM, Riesbeck K. *Haemophilus influenzae* protein E recognizes the C-terminal domain of vitronectin and modulates the membrane attack complex. Mol Microbiol. 2011;81: 80–98. 10.1111/j.1365-2958.2011.07678.x 21542857

[37] Kostrzynska M, Paulsson M, Schmidt KH, Wadström T. Comparative studies on binding of vitronectin and fibronectin to groups A and C streptococci. Microbios. 1992;71: 179–192. 1282649

[38] Li DQ, Lundberg F, Ljungh A null. Characterization of vitronectin-binding proteins of *Staphylococcus epidermidis* . Curr Microbiol. 2001;42: 361–367. 10.1007/s002840010230 11400058

[39] Liang OD, Flock JI, Wadström T. Isolation and characterisation of a vitronectin-binding surface protein from *Staphylococcus aureus* . Biochim Biophys Acta. 1995;1250: 110–116. 7612648 10.1016/0167-4838(95)00076-7

[40] Tyriak I, Ljungh S. Binding of extracellular matrix molecules by enterococci. Curr Microbiol. 2003;46: 435–442. 10.1007/s00284-002-3879-2 12732951

[41] Zipfel PF, Skerka C. *Staphylococcus aureus*: the multi headed hydra resists and controls human complement response in multiple ways. Int J Med Microbiol IJMM. 2014;304: 188–194. 10.1016/j.ijmm.2013.11.004 24461453

[42] Akesson P, Sjöholm AG, Björck L. Protein SIC, a novel extracellular protein of *Streptococcus pyogenes* interfering with complement function. J Biol Chem. 1996;271: 1081–1088. 8557634 10.1074/jbc.271.2.1081

[43] Kurosu T, Chaichana P, Yamate M, Anantapreecha S, Ikuta K. Secreted complement regulatory protein clusterin interacts with dengue virus nonstructural protein 1. Biochem Biophys Res Commun. 2007;362: 1051–1056. 10.1016/j.bbrc.2007.08.137 17825259

[44] Li DQ, Ljungh A. Binding of human clusterin by *Staphylococcus epidermidis* . FEMS Immunol Med Microbiol. 2001;31: 197–202. 11720815 10.1111/j.1574-695X.2001.tb00520.x

[45] Partridge SR, Baker MS, Walker MJ, Wilson MR. Clusterin, a putative complement regulator, binds to the cell surface of *Staphylococcus aureus* clinical isolates. Infect Immun. 1996;64: 4324–4329. 8926106 10.1128/iai.64.10.4324-4329.1996 PMC174374

[46] Liang OD, Rosenblatt S, Chhatwal GS, Preissner KT. Identification of novel heparin-binding domains of vitronectin. FEBS Lett. 1997;407: 169–172. 9166893 10.1016/s0014-5793(97)00330-x

[47] Sheehan M, Morris CA, Pussell BA, Charlesworth JA. Complement inhibition by human vitronectin involves non-heparin binding domains. Clin Exp Immunol. 1995;101: 136–141. 7542572 10.1111/j.1365-2249.1995.tb02289.x PMC1553293

[48] Bergmann S, Lang A, Rohde M, Agarwal V, Rennemeier C, Grashoff C, et al Integrin-linked kinase is required for vitronectin-mediated internalization of *Streptococcus pneumoniae* by host cells. J Cell Sci. 2009;122: 256–267. 10.1242/jcs.035600 19118218

[49] Hallström T, Blom AM, Zipfel PF, Riesbeck K. Nontypeable *Haemophilus influenzae* protein E binds vitronectin and is important for serum resistance. J Immunol Baltim Md 1950. 2009;183: 2593–2601. 10.4049/jimmunol.0803226 19635912

[50] Lopez CM, Wallich R, Riesbeck K, Skerka C, Zipfel PF. *Candida albicans* uses the surface protein Gpm1 to attach to human endothelial cells and to keratinocytes via the adhesive protein vitronectin. PloS One. 2014;9: e90796 10.1371/journal.pone.0090796 24625558 PMC3953207

[51] Voss S, Hallström T, Saleh M, Burchhardt G, Pribyl T, Singh B, et al The choline-binding protein PspC of *Streptococcus pneumoniae* interacts with the C-terminal heparin-binding domain of vitronectin. J Biol Chem. 2013;288: 15614–15627. 10.1074/jbc.M112.443507 23603906 PMC3668722

