# Supplementary material for: Pseudomonas aeruginosa Uses Dihydrolipoamide Dehydrogenase (Lpd) to Bind to the Human Terminal Pathway Regulators Vitronectin and Clusterin to Inhibit Terminal Pathway Complement Attack
Source: PLoS One. 2015 Sep 14;10(9):e0137630. doi: 10.1371/journal.pone.0137630 (PMC4569481; doi:10.1371/journal.pone.0137630)
Supplement: S1 Fig — A, Human serum was depleted from vitronectin by affinity chromatography using rabbit anti-human vitronectin. NHS was incubated with the antibody-coated Sepharose, followed by centrifugation to collect the depleted serum. The vitronectin-depleted serum (HSΔVn) was analyzed for the presence of vitronectin by Western blotting using polyclonal vitronectin antiserum. B, Densitometry measurements revealed a 80% depletion of vitronectin. The mean values of three independent experiments and SD are presented. Statistical significance of differences was estimated using Student’s t test. ***, p≤ 0.001. (PPTX) [file pone.0137630.s001.pptx]

## Slide 1
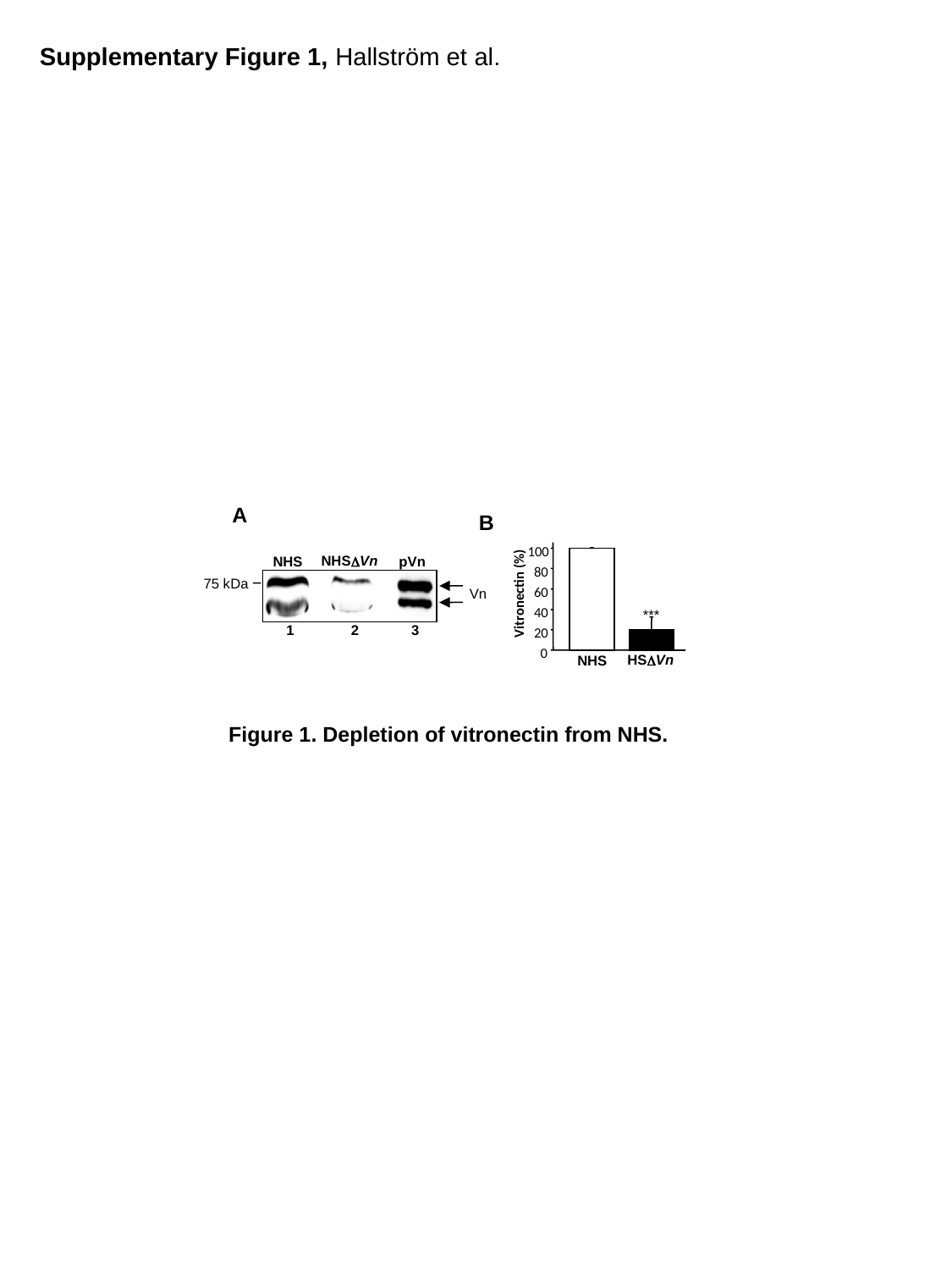

Supplementary Figure 1, Hallström et al.
A
B
100
80
60
Vitronectin (%)
***
40
20
0
HSΔVn
NHS
NHSΔVn
NHS
pVn
75 kDa
Vn
1
2
3
Figure 1. Depletion of vitronectin from NHS.
