# Supplementary material for: Pseudomonas aeruginosa Uses Dihydrolipoamide Dehydrogenase (Lpd) to Bind to the Human Terminal Pathway Regulators Vitronectin and Clusterin to Inhibit Terminal Pathway Complement Attack
Source: PLoS One. 2015 Sep 14;10(9):e0137630. doi: 10.1371/journal.pone.0137630 (PMC4569481; doi:10.1371/journal.pone.0137630)
Supplement: S2 Fig — Recombinant full length Lpd and Lpd deletion mutants were expressed as a N-terminal His-tag protein. The Lpd encoding cDNA was amplified from genomic DNA derived from P. aeruginosa strain PAO1 by PCR, cloned into expression vector pET200D and the corresponding protein was recombinantly expressed in E. coli with a N-terminal His-tag. Recombinant Lpd deletion mutants were purified by affinity chromatography. The purified proteins were separated by SDS-PAGE and analyzed by silver staining. The mobility of the size markers are indicated on the left in kDa. (PPTX) [file pone.0137630.s002.pptx]

## Slide 1
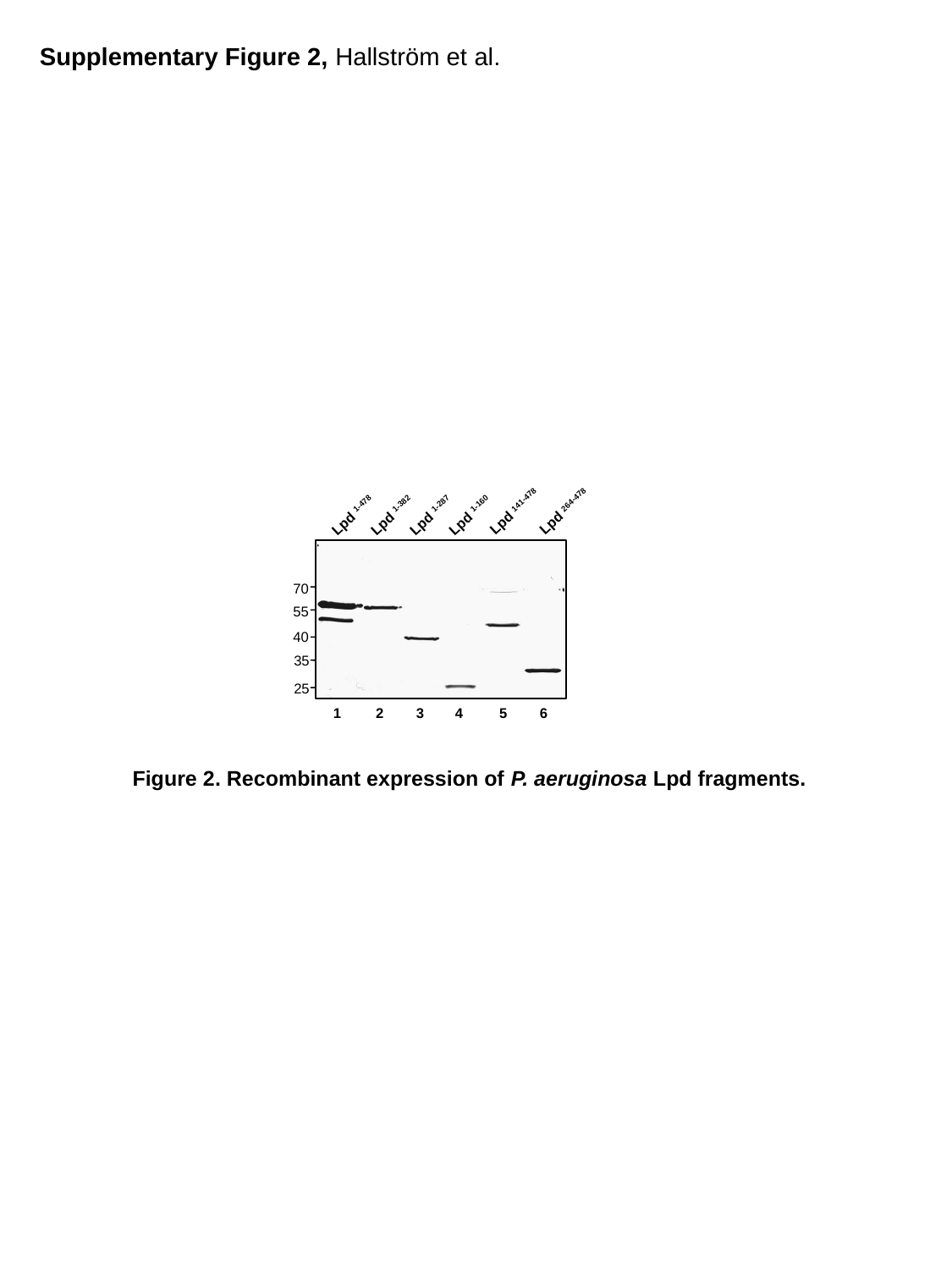

Supplementary Figure 2, Hallström et al.
Lpd 141-478
Lpd 264-478
Lpd 1-478
Lpd 1-382
Lpd 1-287
Lpd 1-160
70
55
40
35
25
1
2
3
4
5
6
Figure 2. Recombinant expression of P. aeruginosa Lpd fragments.
