# Supplementary material for: Pseudomonas aeruginosa Uses Dihydrolipoamide Dehydrogenase (Lpd) to Bind to the Human Terminal Pathway Regulators Vitronectin and Clusterin to Inhibit Terminal Pathway Complement Attack
Source: PLoS One. 2015 Sep 14;10(9):e0137630. doi: 10.1371/journal.pone.0137630 (PMC4569481; doi:10.1371/journal.pone.0137630)
Supplement: S3 Fig — A, Effect of increasing vitronectin levels in the presence of a constant concentration of Factor H (molar ratios are shown). Binding of vitronectin (used at the indicated concentrations) and Factor H (5 μg/ml) to immobilized Lpd was analysed by ELISA. Bound vitronectin was detected with vitronectin antiserum (■) and bound Factor H was detected with Factor H antiserum(◇). B, In a reverse setting, the vitronectin concentration was kept constant (5 μg/ml) and binding of Factor H (used at the indicated concentrations) was evaluated (molar ratios are shown). C, Effect of increasing vitronectin levels in the presence of a constant concentration of plasminogen. Binding of vitronectin (used at the indicated concentrations) and plasminogen (5 μg/ml) to immobilized Lpd was analysed by ELISA (molar ratios are shown). Bound vitronectin was detected with vitronectin antiserum (■) and bound plasminogen was detected with plasminogen antiserum (◇). D, In a reverse setting, the vitronectin concentration was kept constant (5 μg/ml) and binding of plasminogen (used at the indicated concentrations) was evaluated (molar ratios are shown). The mean values of three independent experiments and SD are presented. Statistical significance of differences was estimated using Student’s t test. *, p≤ 0.05. (PPTX) [file pone.0137630.s003.pptx]

## Slide 1
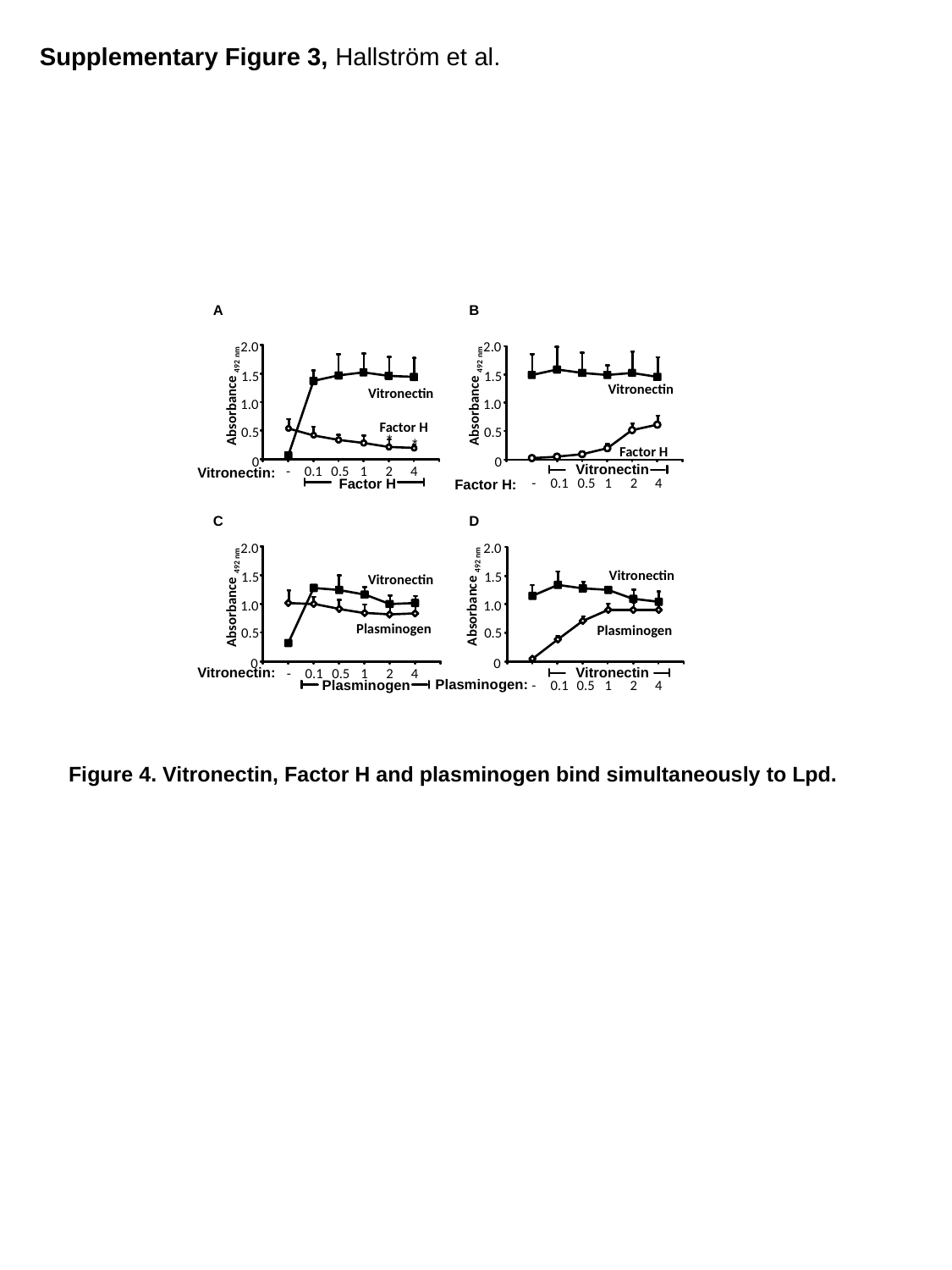

Supplementary Figure 3, Hallström et al.
B
A
2.0
2.0
1.5
1.5
Vitronectin
Vitronectin
Absorbance 492 nm
Absorbance 492 nm
1.0
1.0
Factor H
0.5
0.5
*
*
Factor H
0
0
Vitronectin
Vitronectin:
-
4
0.1
0.5
1
2
Factor H
Factor H:
-
0.1
0.5
1
2
4
C
D
2.0
2.0
 Vitronectin
1.5
1.5
 Vitronectin
Absorbance 492 nm
Absorbance 492 nm
1.0
1.0
 Plasminogen
 Plasminogen
0.5
0.5
0
0
Vitronectin:
Vitronectin
-
4
0.1
0.5
1
2
Plasminogen:
Plasminogen
-
0.1
0.5
1
2
4
Figure 4. Vitronectin, Factor H and plasminogen bind simultaneously to Lpd.
